# The potential of ME1 in guiding immunotherapeutic strategies for ovarian cancer: insights from pan-cancer research

**DOI:** 10.3389/fimmu.2025.1571842

**Published:** 2025-05-29

**Authors:** Jiahui Wei, Ming Wang, Shuiqing Xu, Paizigul Tusufuhan, Yumei Wu

**Affiliations:** ^1^ Department of Gynecologic Oncology, Beijing Obstetrics and Gynecology Hospital, Capital Medical University. Beijing Maternal and Child Health Care Hospital, Beijing, China; ^2^ Beijing Chaoyang Hospital, Capital Medical University, Beijing, China; ^3^ Department of Biochemistry and Molecular Biology, School of Basic Medical Sciences, Capital Medical University, Beijing, China

**Keywords:** ME1, pan-cancer analysis, tumor immune microenvironment, ovarian cancer, immunotherapy

## Abstract

**Objective:**

ME1 catalyzes the conversion of malic acid into acetic acid, thereby linking glucose metabolism to the citric acid cycle. In recent years, the role of ME1 in various tumors has only been superficially explored. Therefore, our objective is to analyze the potential functions of ME1 in pan-cancer, with a particular focus on its role in ovarian cancer.

**Methods:**

We analyzed the ME1 expression levels in both normal and tumor tissues across various cancer types. CBIOPORTAL was utilized to assess the mutation frequency and specific sites of ME1. Additionally, we examined the correlation between ME1 expression and several factors, including methylation status, tumor mutation burden (TMB), microsatellite instability (MSI), immune regulator genes, immune checkpoints, tumor microenvironment scores, functional enrichment, single-cell analysis, and drug sensitivity. The Estimate Algorithm assessed the correlation between ME1 expression and the tumor immunochemical microenvironment. Small interfering RNA and chronic viruses were utilized to downregulate and upregulate ME1 expression in two ovarian cancer cell lines, respectively, to conduct experiments on cell proliferation and migration.

**Results:**

Our results revealed that ME1 exhibited disorders across various tumors, with the predominant form of genetic mutation identified being a missense mutation. Among the various tumors analyzed, ME1 demonstrated a significant correlation with methylation levels, TMB, MSI, immune checkpoints, immunomodulatory regulatory genes, tumor microenvironment scores, and immune infiltration. Functional enrichment analysis and single-cell analysis indicated that ME1 expression was associated with metabolic regulation, macrophage immune responses, antioxidant defense mechanisms, and the potential tumor microenvironment. The elevated levels of ME1 may be associated with a more favorable response to specific immunotherapy, suggesting that ME1 has potential applications in guiding immunotherapeutic strategies. *In vitro* research results demonstrated that in ovarian cancer cell lines, the knockdown of ME1 inhibited the proliferation and migration of tumor cells. Conversely, the overexpression of ME1 appeared to promote tumor cell proliferation and migration.

**Conclusions:**

ME1, a metabolic-related factor, has the potential to serve as a biomarker for tumor progression and immune infiltration, particularly in ovarian cancer. It may signify a metabolic reprogramming that supplies energy for tumor progression and immunotherapy, offering valuable insights for the development of personalized therapies.

## Introduction

Cancer is a multifaceted disease that affects millions of individuals globally and is on the rise (1). Advances in medical treatment have led to various therapeutic approaches, including surgical intervention, chemotherapy, radiotherapy, immune-targeted therapy, and combination therapies (2, 3). However, due to the intricate pathogenesis of tumors and the individual differences among patients, some individuals continue to experience recurrence, drug resistance, and adverse side effects, all of which adversely impact survival and quality of life (4, 5). Ovarian cancer is among the most lethal malignancies affecting the female reproductive system, with over 300,000 new cases reported globally each year and a five-year survival rate of less than 50% (6). Due to its insidious early symptoms, approximately 70% of patients are diagnosed at an advanced stage (III/IV). Although surgery combined with platinum-based chemotherapy is the standard treatment, the recurrence rate of drug resistance is as high as 80%, and there is a lack of effective targeted therapeutic strategies (7). In recent years, immunotherapy has demonstrated potential in the treatment of ovarian cancer; however, the overall response rate remains below 20% (8). Consequently, investigating key metabolic regulatory molecules within the ovarian cancer microenvironment may yield breakthroughs for the development of novel combination immunotherapies.

Malic enzyme 1 (ME1) is a cytoplasmic protein that catalyzes the conversion of malate to pyruvate, concurrently generating NADPH from NADP. Early research identified ME1 as a key mediator of intermediary metabolism, primarily by its involvement in lipid and cholesterol biosynthesis. ME1 is implicated in the development of various tumors, with multiple studies documenting its oncogenic properties in numerous epithelial cancers (9). Its overexpression contributes to the growth and metastasis of gastric cancer cells by depleting NADPH and inducing elevated levels of reactive oxygen species (ROS) (10). In the context of oral cancer, the upregulation of ME1 is closely associated with poor prognosis (11). Conversely, the knockdown of ME1 expression inhibits cell proliferation and migration. Furthermore, ME1 expression significantly enhances the growth and invasion of cancer cells in basal-like breast cancer (12). Zhang et al (13). found that elevated levels of ME1 are associated with a poorer prognosis in patients with cytogenetically normal acute myeloid leukemia (CN-AML) and may facilitate cancer growth through specific pathways. Studies have demonstrated that ME1 expression levels influence cancer susceptibility to oxidative phosphorylation (OXPHOS) inhibitors, with elevated ME1 expression diminishing the synthetic lethality between the pentose phosphate pathway (PPP) and OXPHOS. Synthetic lethality, defined as a lethal interaction between two pathways when simultaneously inhibited (e.g., PPP and OXPHOS), can be exploited therapeutically. Elevated ME1 expression may diminish this synthetic lethality by compensating for metabolic vulnerabilities through NADPH production or lipid synthesis, thereby reducing the efficacy of OXPHOS inhibitors in combination with PPP-targeted therapies. Studies have identified the ME1 gene as a novel immunometabolic enzyme target for NF-κB signaling in innate immune cells. The upregulation of its expression activates the immune NADPH pool (i.e., the cellular reservoir of NADPH), thereby facilitating inflammatory responses and contributing to the pathogenesis of inflammatory diseases such as rheumatoid arthritis and systemic lupus erythematosus (14). Some studies have indicated that the expression of ME1 in the endothelium is crucial in the context of pulmonary hypertension. Inhibition of ME1 leads to alterations in the malate-aspartate NADH (nicotinamide adenine dinucleotide hydrogenation) shuttle, which occur in an ATP-dependent manner. This process subsequently activates adenosine production, thereby facilitating a balance between oxidative phosphorylation and glycolysis (15). Metabolic reprogramming is a fundamental characteristic of ovarian cancer progression (16). Research indicates that ovarian cancer cells exhibit a strong dependence on NADPH for maintaining redox balance and facilitating lipid synthesis (17). As a principal source of NADPH, ME1 may affect therapeutic responses by regulating the immunosuppressive microenvironment, including the modulation of macrophage polarization and T cell exhaustion.

In summary, ME1 is a pivotal functional metabolic enzyme that significantly contributes to the metabolism of both normal and cancer cells. It plays an essential role in lipogenesis and steroidogenesis, thereby influencing the overall metabolic processes of lipid and steroid synthesis. Although the importance of pan-cancer analysis in understanding tumorigenesis and progression is self-evident, research on the role of ME1 across various cancers is notably insufficient, particularly regarding its role in ovarian cancer, which remains largely unexplored. Therefore, this study aims to examine the fundamental expression, methylation levels, tumor mutation burden, immune microenvironment, tumor cell stemness, and immune-related functions of ME1 in a pan-cancer context through bioinformatics research. Simultaneously, we are conducting experimental validation of cellular functions in ovarian cancer cell lines to further elucidate the role of ME1 in the development and progression of ovarian cancer, with the hope of providing new insights for its diagnosis and treatment.

## Materials and methods

### Pan-cancer landscape of ME1

#### Basic expression analysis

The Tumor Immune Estimation Resource version 2 (TIMER2, http://timer.cistrome.org/), the Gene Expression Profiling Interactive Analysis (GEPIA, http://gepia.cancer-pku.cn/), and the Gene Expression database of Normal and Tumor tissues (GENT2, http://gent2.appex.kr/gent2) web databases were utilized to validate the differences in ME1 expression between tumor and non-tumor tissues across various cancer types from multiple perspectives. The distribution of gene expression in different organs was visualized using the ‘Interactive Bodymap’ module in GEPIA.

#### Protein analysis

To assess differences in ME1 protein expression, we analyzed immunohistochemistry (IHC) images of normal and tumor tissues, including lung, liver, cervical, and thyroid cancers, utilizing data from the Human Protein Atlas (HPA) (http://www.proteinatlas.org/). Furthermore, ME1 protein expression levels in additional cancer types were corroborated using the CPTAC database accessed through the UALCAN portal (http://ualcan.path.uab.edu/analysis-prot.html).

#### Mutation and methylation analysis

Data on mutations and DNA copy number variations of ME1 across different cancers were sourced from the cBioPortal database (http://www.cbioportal.org), employing the mutation mapper tool to visualize the distribution of these mutations. To explore the relationship between ME1 expression and DNA methylation across 33 cancer types available in the TCGA database, we utilized the SMART platform (http://www.bioinfo-zs.com/smartapp/), an interactive web application designed for comprehensive DNA methylation analysis and visualization (18). We input ‘ME1’ into the ‘Quick Start’ module, subsequently utilizing the ‘CpG-aggregated methylation’ module to calculate the methylation levels of ME1 across various cancer types, with the results depicted in a box plot. RNA methylation influences the processing, translation, and degradation of RNA, regulates the tumor microenvironment, and consequently impacts the physiological and pathological processes of cancer cells (19). The correlation between the ME1 gene and RNA methylation genes was analyzed using the Sangerbox 3.0 website (http://sangerbox.com/home.html), and a heatmap was generated to visualize the results.

#### Correlations of ME1 expression with tumor mutation burden and microsatellite instability

Tumor mutational burden (TMB) is an emerging biomarker that has gained increasing attention for its potential role in predicting the efficacy of tumor immunotherapy. Microsatellite instability (MSI) is frequently utilized as a marker and holds significant importance in cancer diagnosis and prognosis. Spearman’s correlations were employed to analyze the associations between MEI, TMB, and MSI.

#### Functional enrichment analysis

The STRING database (https://string-db.org/) provided ME1-interacting proteins for the analysis of protein-protein interaction networks (20). To further investigate the biological function of ME1, we utilized LinkedOmics (www.linkedomics.org/login.php), a robust platform designed for acquiring, analyzing, and comparing multi-omics cancer data across various tumor types (21, 22). Additionally, the Pearson correlation test was employed to establish the correlation between ME1 and co-expressed genes. Subsequently, enriched results were obtained by selecting the “Enrichment Analysis” and “KEGG pathway” options within the “LinkInterpreter” module (23).

#### Single-cell RNA sequencing data analysis

CancerSEA (http://biocc.hrbmu.edu.cn/CancerSEA/home.jsp) is a multifunctional website designed to present a comprehensive atlas of functional states in cancer at the single-cell level, encompassing 14 distinct functional states: stemness, invasion, metastasis, proliferation, epithelial-mesenchymal transition, angiogenesis, apoptosis, cell cycle progression, differentiation, DNA damage, DNA repair, hypoxia, inflammation, and quiescence. This atlas is based on an analysis of 41,900 single cancer cells derived from 25 different cancer types (24). The correlation data between ME1 expression and different tumor functions based on single-cell sequencing data were analyzed (3). Furthermore, we utilized the Tumor Immune Single-cell Hub (TISCH) database (http://tisch.comp-genomics.org/home/) to analyze the expression of ME1 across various cancers, including ovarian cancer, breast invasive carcinoma, and non-small cell lung cancer at the single-cell level.

#### Immune regulatory gene, immune checkpoints, and tumor stemness score analysis

Immune regulatory genes, immune checkpoints, and tumor stemness scores are pivotal concepts in the realms of contemporary cancer immunotherapy and tumor biology research. These elements are intricately interconnected with tumor immune evasion, the tumor microenvironment, treatment resistance, and the characteristics of cancer stem cells (25, 26). In this study, we employed the Sangerbox 3.0 platform to explore the relationship between ME1 gene expression and five immune pathway markers, including chemokines, receptors, MHC, immunoinhibitors, and immunostimulators. Additionally, we examined two categories of immune checkpoint pathway genes (inhibitory and stimulatory) alongside tumor stemness scores, with the results visualized through heat maps.

#### Immune cell infiltration analysis

The analysis of immune cell infiltration serves as a significant tool for comprehending the tumor microenvironment and its influence on cancer progression and therapeutic responses. In this study, the ESTIMATE algorithm was utilized to evaluate the relationship between ME1 expression in various cancers, focusing on immune, stromal, and tumor purity scores. Additionally, the TIMER database was employed to explore the association between ME1 expression and the levels of different immune cell infiltrates across all cancers in The Cancer Genome Atlas (TCGA). To further investigate the potential signaling pathways linking ME1 to immune infiltration, we focused on ovarian cancer as a case study, dividing ovarian cancer patients into high-expression and low-expression groups based on the median expression of ME1. We used the CIBERSORT algorithm to analyze the composition of tumor-infiltrating immune cells and to compare the differences in immune cell proportions between high-risk and low-risk groups. Furthermore, the ESTIMATE algorithm was further employed to assess differences in immune, stromal, and tumor purity scores between the two risk groups. To further investigate the biological functions and pathways associated with ME1 in ovarian cancer, Gene Set Enrichment Analysis (GSEA) was employed to assess differences in enrichment.

#### Drug sensitivity analysis and immunotherapy analysis

The effectiveness of targeted therapy was predicted using the ‘pRRophetic’ package, while the semi-maximum inhibitory concentration index (IC50) was employed to measure the degree of tumor tolerance to the drug in ovarian cancer patients (19). The kmplot (https://kmplot.com/analysis/) was utilized to analyze the associations between immune checkpoint blockade (ICB) treatments and ME1 (22).

### Experimental validation of ME1 in ovarian cancer

#### Cell culture

The human ovarian cancer cell line A2780 and the normal ovarian cell line IOSE80 were obtained from BeNa Culture Collection (Henan, China) and cultured in RPMI 1640 medium supplemented with 10% fetal bovine serum, 100 U/ml penicillin, and 100 μg/ml streptomycin (Procell, Wuhan, China). The human ovarian cancer cell lines OVCAR-3 was sourced from Wuhan Procell Life Science & Technology Co., Ltd. (Wuhan, China). OVCAR-3 was cultured in RPMI 1640 medium supplemented with 0.01 mg/ml insulin, 20% fetal bovine serum, and 1% penicillin/streptomycin (Procell, Wuhan, China). All cell lines were maintained at 37°C in a humidified incubator with 5% CO2.

#### RNA extraction and quantitative real-time PCR analysis

Total RNA was extracted using the Total RNA Extraction Kit (Beijing Solebo Technology, Beijing, China). cDNA synthesis was performed with the RevertAid RT kit (Thermo Fisher Scientific, Beijing, China). Reverse transcription polymerase chain reaction (RT-PCR) was conducted using the SYBR Green assay (Beijing Qihangxing Biotechnology, Beijing, China) on an AB 7500 machine (Applied Biosystems Inc., USA). The SYBR primers utilized in this study are listed in **Supplementary Table S1**. GAPDH was employed as an internal control for normalization. The relative RNA abundance (fold change) of each long non-coding RNA (lncRNA) was calculated using the standard 2−ΔΔCT method. Each sample was analyzed in triplicate.

#### Western blotting

The entire protein extraction kit (Solebao, Beijing, China) was utilized to extract intracellular proteins, while the BCA protein detection kit (Plitely, Beijing, China) was employed to determine the protein concentration in the samples. Protein samples were then subjected to electrophoresis on a 10% SDS-polyacrylamide gel. Following this, the proteins were transferred to polyvinylidene fluoride (PVDF) membranes. The membranes were blocked with 5% skim milk at 37°C for 2 hours, after which primary and enzyme-labeled secondary antibodies were added and incubated sequentially. Finally, the target protein was visualized using an ECL ultra-sensitive chemiluminescence detection kit (Yamei, Beijing, China). The antibodies used included: primary antibodies rabbit anti-ME1 (Abcam, UK) and mouse anti-GAPDH (Abcam, UK); secondary antibodies goat anti-rabbit IgG (Abcam, UK) and goat anti-mouse IgG (Abcam, UK).

#### Small interfering RNA transfection and lentivirus transfection

Small interfering RNAs (siRNAs) targeting ME1, along with non-targeting negative controls (NC), were procured from Shanghai GenePharma Co., Ltd. and subsequently transfected into the human ovarian cancer cell lines A2780 and OVCAR3 using Lipofectamine 3000 (Invitrogen), in accordance with the manufacturer’s instructions. After 72 hours of transfection, total mRNA and protein were extracted to evaluate the transfection efficiency of the siRNA. The sequences of the ME1 siRNAs are presented in **Supplementary Table S2**. Additionally, ME1 overexpression lentivirus and the negative control virus, which contains GFP and puromycin resistance genes, were obtained from Shanghai Genechem Co., Ltd. Human ovarian cancer cell lines A2780 and OVCAR3 were cultured in 12-well plates. Once the cells reached 50% confluence, lentiviral transfection (vector: GV492 Ubc-MCS-3FLAG-CBh-gcGFP-IRES-puromycin) was performed at a multiplicity of infection (MOI) of 30, followed by puromycin selection for screening to establish a human ovarian cancer cell line that stably overexpresses ME1. The overexpression efficiency of ME1 was verified using western blotting (WB).

#### CCK8 assay

Human ovarian cancer cell lines A2780 and OVCAR3, subjected to ME1 knockdown and overexpression, were seeded into a 96-well plate at a density of 7,000 cells per well. Each well was supplemented with 100 µL of culture medium and cultured in an incubator. In both the experimental and control groups, CCK8 reagent was added at specific time points (0 h, D1, D2, D3, D4, D5, and D6) in accordance with the manufacturer’s instructions. Following a 2-hour incubation at 37°C, the absorbance at a wavelength of 450 nm (optical density value) was measured using a microplate reader. Subsequently, a cell proliferation curve was generated to analyze cell proliferation.

#### Clone formation assay

Cell cloning experiments were conducted to assess the proliferative capacity of cells. Human ovarian cancer cell lines (A2780 and OVCAR3) with either ME1 knockdown or overexpression were seeded into six-well plates at a density of 1500 cells per well, followed by the addition of 2 mL of culture medium. The cells were cultured in an incubator until distinct colonies formed. Subsequently, the cells were fixed with 4% paraformaldehyde for 20 minutes, stained with 0.1% crystal violet at room temperature for 2 hours, and then photographed and counted. A higher number of clones indicates a greater ability for cell proliferation.

#### Cell scratch assay

Human ovarian cancer cell lines A2780 and OVCAR3, subjected to ME1 knockdown and overexpression, were seeded in six-well plates. Once the cultures reached approximately 90% confluency, vertical scratch wounds were created using a 200 μL pipette tip. Observations and photographs were taken using an optical microscope at 100× magnification at both 0 and 24 hours. ImageJ software was utilized to analyze scratch mobility. The cell migration rate was calculated using the formula: (scratch area at 0 h - scratch area at 24 h)/scratch area at 0 h × 100%.

#### Transwell assay

The human ovarian cancer cell lines A2780 and OVCAR3, which were subjected to either knockdown or overexpression of ME1, were collected using serum-free medium. These cells were then inoculated into the upper chamber of a polycarbonate membrane with a pore size of 8.0 μm at a concentration of 6 × 10^5 cells/mL. Subsequently, 500 μL of either A2780 complete medium or OVCAR3 complete medium was added to the lower chamber, and the assembly was incubated at 37°C for 48 hours. After incubation, the original medium was discarded, and the lower chamber was fixed in 4% paraformaldehyde for 20 minutes. Following fixation, the lower chamber was stained with a 0.1% crystal violet staining solution for 2 hours. The crystal violet solution was then aspirated from the well, and the chamber was rinsed repeatedly with water. A cotton swab was used to remove any remaining cells from the upper chamber, which was then allowed to dry in a ventilated area. Finally, images were captured under a microscope, and the number of migrated cells was quantified using Image J software.

#### Statistical analysis

For this study, all data were calculated, graphed, and statistically analyzed using GraphPad Prism 8.0 and R software (version 3.6.3). The statistical analyses for qPCR, clone formation, CCK8, cell scratch, and transwell data were performed using Student’s t-test or one-way ANOVA. Significance levels were defined as *p* < 0.05 (*), *p* < 0.01 (**), and *p* < 0.001 (***).

## Results

### Pan-cancer landscape of ME1 expression and genetic alterations

#### ME1 expression analysis in pan-cancer

**Figure 1:**
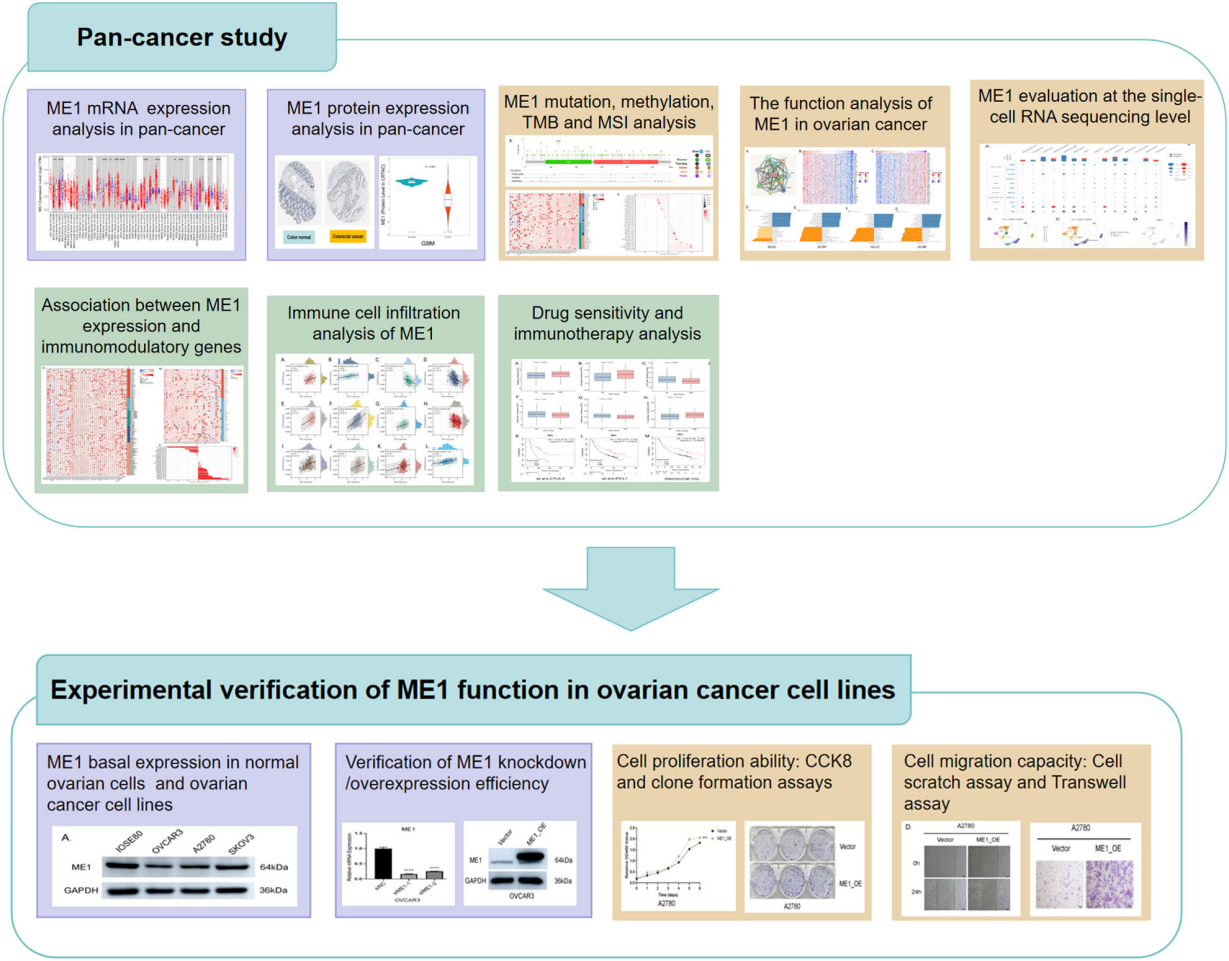
Flowchart of study design.

**Figure 2:**
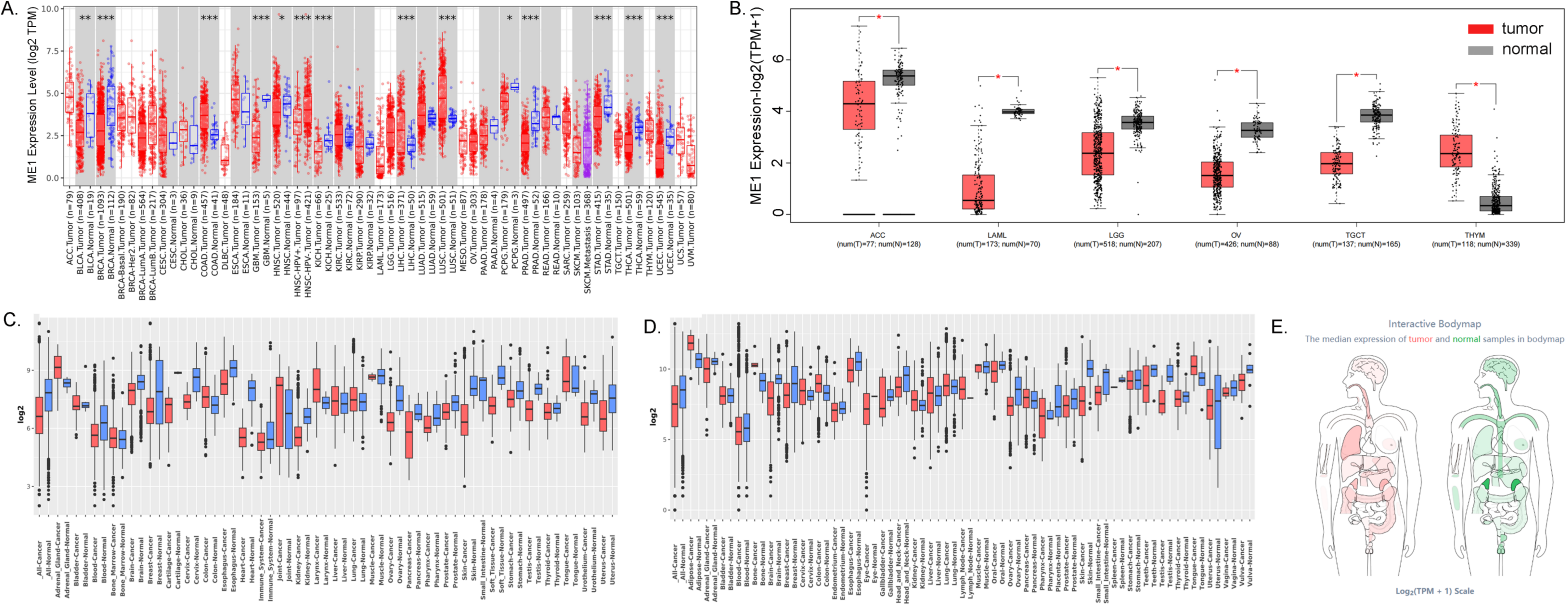
ME1 expression analysis in pan-cancer. **(A)** Differences in ME1 expression between 33 normal and tumor tissues in TCGA from the TIMER2.0 database. **(B)** Differences in ME1 expression between cancers from the TCGA database and normal samples from the GTEx database. **(C, D)** ME1 expression level in GENT2 GPL96 and GPL570. **(E)** Expression and distribution of ME1 in various organs. The left panel depicts the expression of ME1 in the corresponding tumor tissues, while the right panel represents the expression of ME1 in normal tissues. A darker color indicates a higher expression level of ME1.*p < 0.05, **p < 0.01, ***p < 0.001.

The flowchart illustrating the data collection, categorization, and analysis process is presented in **Figure 1**. To investigate the differential expression of ME1 across various human cancers and their corresponding paracancerous tissues, we utilized the TIMER2.0 platform to analyze RNA sequencing data from 33 cancers within The Cancer Genome Atlas (TCGA) (**Figure 2A**). Additionally, we employed the GEPIA2 platform, in conjunction with the GTEx and TCGA databases, to analyze various cancers within TCGA, excluding normal samples. The results of this analysis are presented in **Figure 2B**. Our findings indicated that ME1 was highly expressed in several cancers, including bladder cancer (BLCA), breast cancer (BRCA), glioblastoma (GBM), head and neck squamous cell carcinoma (HNSC), kidney chromophobe (KICH), pheochromocytoma and paraganglioma (PCPG), prostate cancer (PRAD), stomach cancer (STAD), thyroid cancer (THCA), thymoma (THYM), and uterine corpus endometrial carcinoma (UCEC). In contrast, ME1 shows low expression levels in colorectal cancer (COAD), liver hepatocellular carcinoma (LIHC), lung squamous cell carcinoma (LUSC), adrenocortical carcinoma (ACC), acute myeloid leukemia (LAML), lower-grade glioma (LGG), ovarian cancer (OV), testicular germ cell tumors (TGCT), and other cancers, with statistically significant differences observed. Furthermore, we conducted additional analysis and verification of these results using the GENT2 GPL570 and GPL96 platforms (**Figures 2C, D**). Subsequently, we visualized the expression distribution pattern of ME1 using organ graphs. The left panel illustrates the expression of ME1 in the corresponding tumor tissues, while the right panel represents its expression in normal tissues. A darker color indicates a higher level of ME1 expression (**Figure 2E**).

#### ME1 protein expression levels in pan-cancer

**Figure 3:**
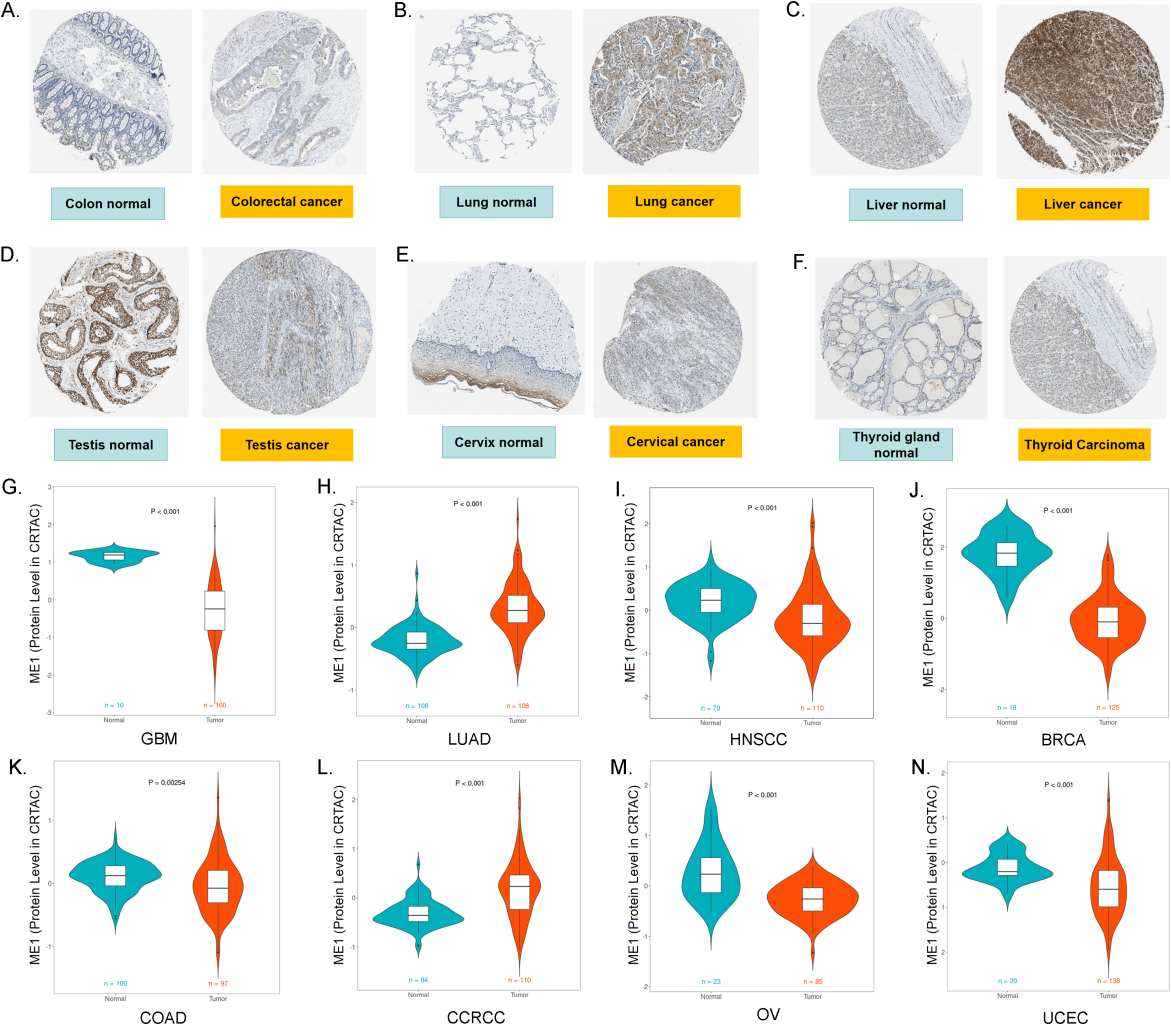
ME1 protein expression levels in pan-cancer. **(A-F)** The expression of ME1 in both tumor and normal tissues, including the colon, lung, liver, testis, cervix, and thyroid gland, was analyzed using data from the HPA database. **(G-N)** ME1 protein expression levels in GBM, LUAD, HNSCC, BRCA, COAD, CCRCC, OV and UCEC from the CPTAC database.

In this study, we demonstrated the expression of ME1 in both tumor and normal tissues, including the colon, lung, liver, testis, cervix, and thyroid gland, utilizing data from the HPA database (**Figures 3A-F**). To enhance the presentation of expression differences in ME1, we employed the CPTAC database to further validate the significance of ME1 expression and the accuracy of our analysis at the protein level. The results are illustrated in violin plots (**Figures 3G-N**). The reproducibility and consistency of these findings have been confirmed across multiple databases, tumor types, various methods, and omics analyses. This suggests that the deregulation of ME1 expression may be implicated in various cancers and is unlikely to be attributed to technical artifacts, randomness, or bias in the sample identification standards within the database.

#### ME1 mutation, methylation, TMB and MSI analysis of ME1 in pan-cancer

**Figure 4:**
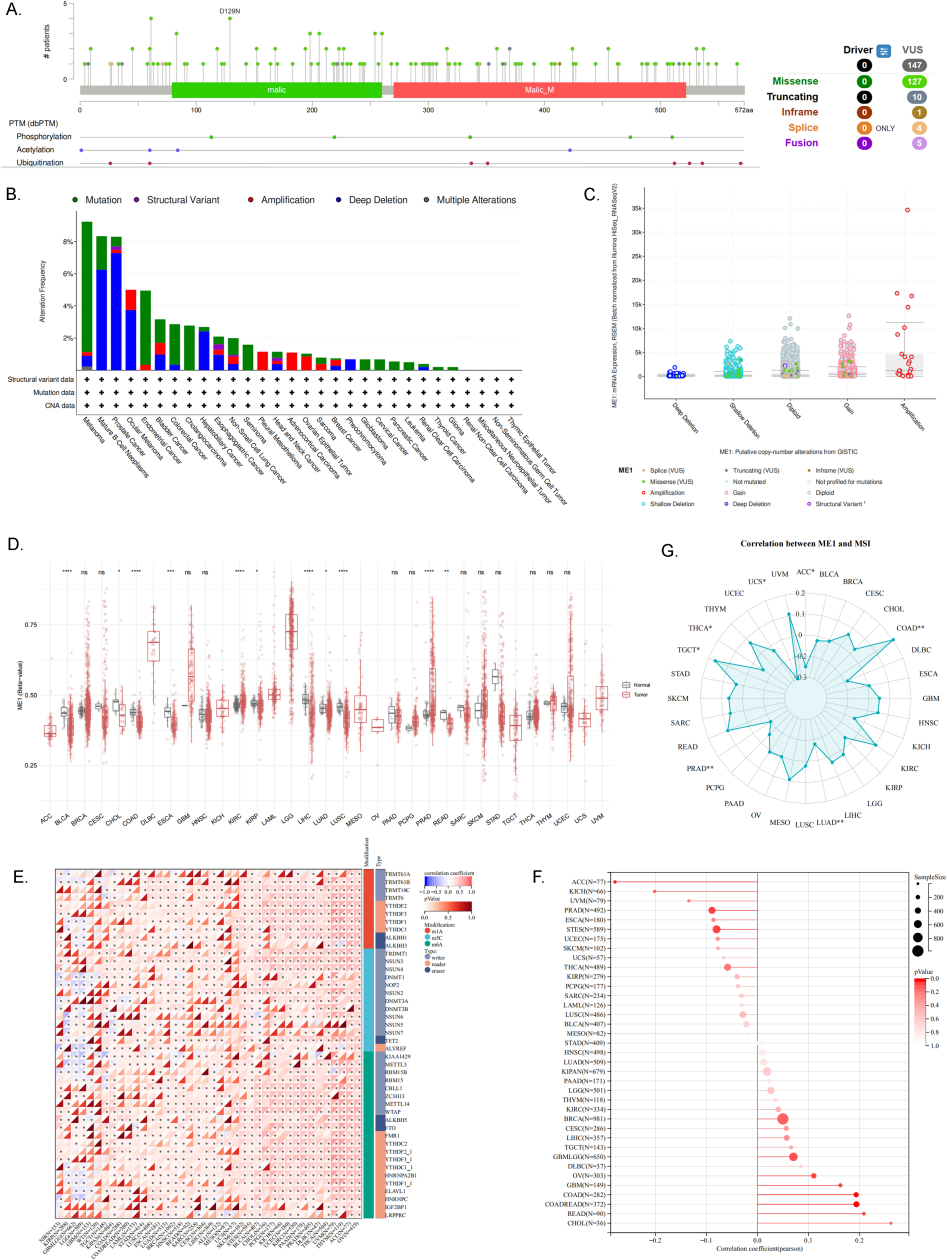
ME1 mutation, methylation, TMB and MSI analysis. **(A)** Distribution of ME1 mutations within protein structural domains. **(B)** Frequency and mutation types of ME1 gene alterations in pan-cancer. **(C)** Correlation between ME1 expression levels and genetic alterations. **(D)** DNA methylation levels of the ME1 gene in pan-cancer. **(E)** Expression levels of the ME1 gene alongside 44 marker genes associated with class III RNA-modified genes [m1A (10), m5C (13), m6A (21)] in each sample. **(F)** Association between ME1 expression and TMB. **(G)** The relationship between ME1 and MSI in multiple types of cancer. Significant negative correlation in 5 cancer types (ACC、LUAD、PRAD、THCA、UCS) and significant positive correlation in 2 cancer types (COAD、TGCT). *p < 0.05, **p < 0.01, ***p < 0.001, ****p < 0.0001. ns, not significant.

Given the significant role of gene mutations in tumor development, we utilized the CBioPortal platform to conduct a comprehensive analysis of ME1 mutations across various pan-cancers. The predominant form of genetic mutation identified in ME1 was a missense mutation (**Figure 4A**). The results indicated that melanoma, mature B-cell neoplasms, and prostate cancer were the three cancer types exhibiting the highest frequency of mutations (**Figure 4B**). Additionally, **Figure 4C** further explored the relationship between ME1 expression and genetic alterations.

Methylation may serve as a promising biomarker for the diagnosis, prognosis, and prediction of cancer. Initially, we utilized the SMART platform to assess the DNA methylation levels of ME1 in tumor tissues compared to their corresponding normal tissues, as sourced from the TCGA database (**Figure 4D**). The results revealed significant differences in ME1 DNA methylation across 11 types of cancer. Notably, ME1 methylation levels were significantly reduced in the tissues of patients with BLCA, cholangiocarcinoma (CHOL), COAD, esophageal carcinoma (ESCA), kidney renal papillary cell carcinoma (KIRP), LIHC, lung adenocarcinoma (LUAD), LUSC, and rectum adenocarcinoma (READ), whereas an increase in methylation levels was observed in kidney renal clear cell carcinoma (KIRC) and PRAD. Subsequently, to further elucidate the potential relationship between ME1 and cancer, we conducted an analysis of the correlation between ME1 and RNA methylation levels, with the results presented in **Figure 4E**.

Recent studies have demonstrated that tumors characterized by high mutational burden (TMB-H) and high microsatellite instability (MSI-H) serve as predictive biomarkers for assessing responses to immune checkpoint blockade therapy (27). In this study, we utilized bubble charts and circle charts to further visualize the correlation between ME1 expression levels and both TMB and MSI (**Figures 4F, G**).

#### ME1 evaluation at the single-cell RNA sequencing level

**Figure 5:**
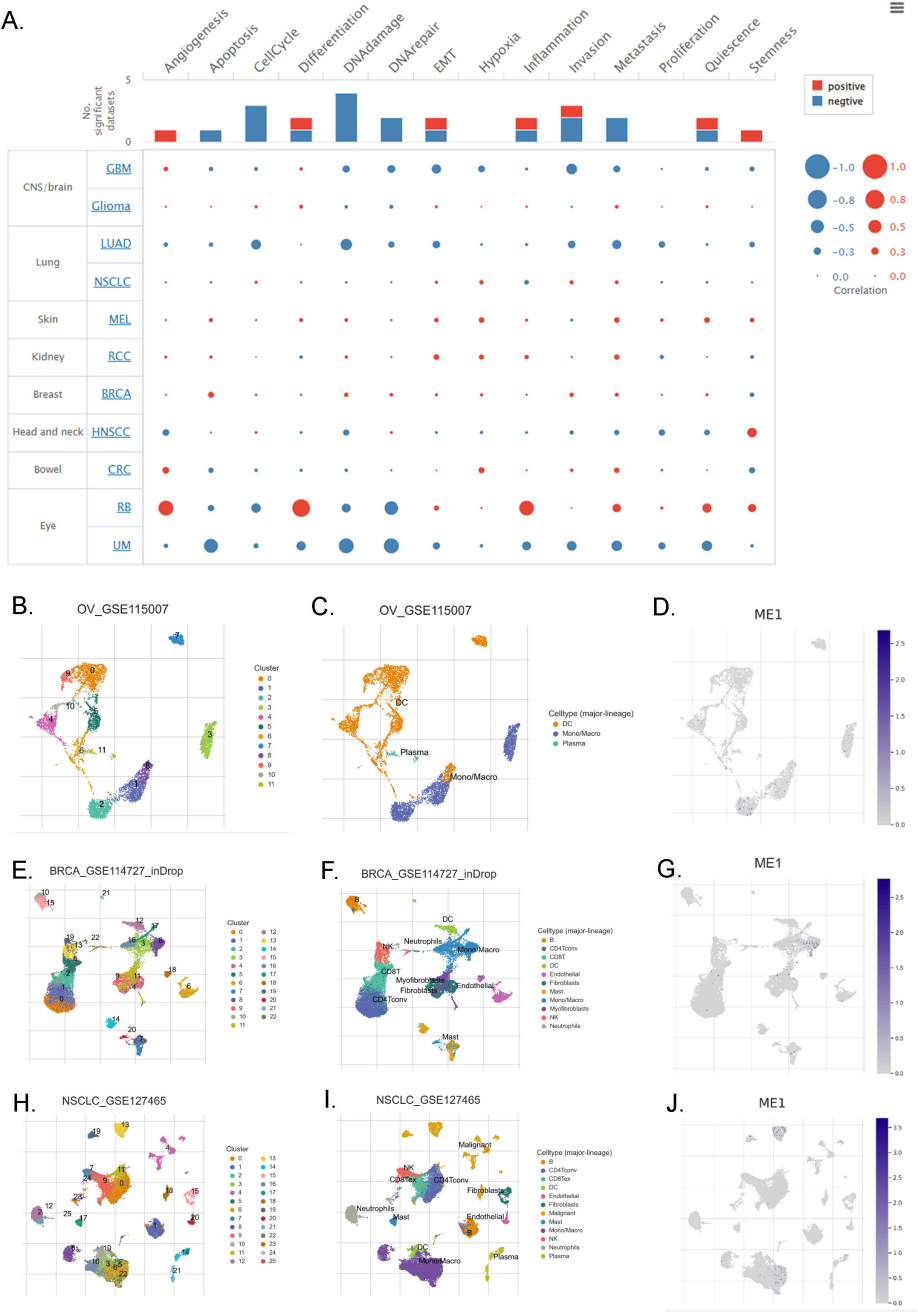
ME1 evaluation at the single-cell RNA sequencing level. **(A)** The correlation between the expression of ME1 and the 14 functional states of cancer across pan-cancer was analyzed. **(B-D)** The identified cell clusters, cell types, and the expression level of ME1 in ovarian cancer tissues based on the OV_GSE115007 dataset. **(E-G)** The identified cell clusters, cell types, and the expression level of ME1 in breast cancer tissues based on the BRCA_GSE114727_inDrop dataset. **(H-J)** The identified cell clusters, cell types, and the expression level of ME1 in non-small cell lung cancer tissues based on the NSCLC_GSE127465 dataset.

The correlation between ME1 and 14 cancer functional states was assessed using single-cell sequence data from CancerSEA (**Figure 5A**). Additionally, we employed the TISCH database to further explore the expression of ME1 at the single-cell RNA sequencing (scRNA-seq) level. Analysis of the scRNA-seq data from the OV_GSE115007 dataset identified 12 cell clusters and 3 cell types within ovarian cancer tissues, with ME1 exhibiting enrichment primarily in monocytes/macrophages (**Figures 5B-D**). Similarly, analysis of the scRNA-seq data from the BRCA_GSE114727_inDrop dataset revealed 23 cell clusters and 11 cell types in breast cancer tissues, where ME1 was enriched in both monocytes/macrophages and myofibroblasts (**Figures 5E-G**). Furthermore, analysis of the scRNA-seq data from the NSCLC_GSE127465 dataset identified 26 cell clusters and 12 cell types in non-small cell lung cancer tissues, with ME1 being enriched in both monocytes/macrophages and malignant cells (**Figures 5H-J**). The elevated expression of ME1 in macrophages may suggest its involvement in metabolic regulation, immune response, antioxidant defense, immune tolerance, and potentially the tumor microenvironment. This enrichment may assist macrophages in managing various immune and metabolic challenges by supporting their energy requirements and redox status. Further validation through additional clinical trials is necessary.

#### Association between ME1 expression and immunomodulatory genes

**Figure 6:**
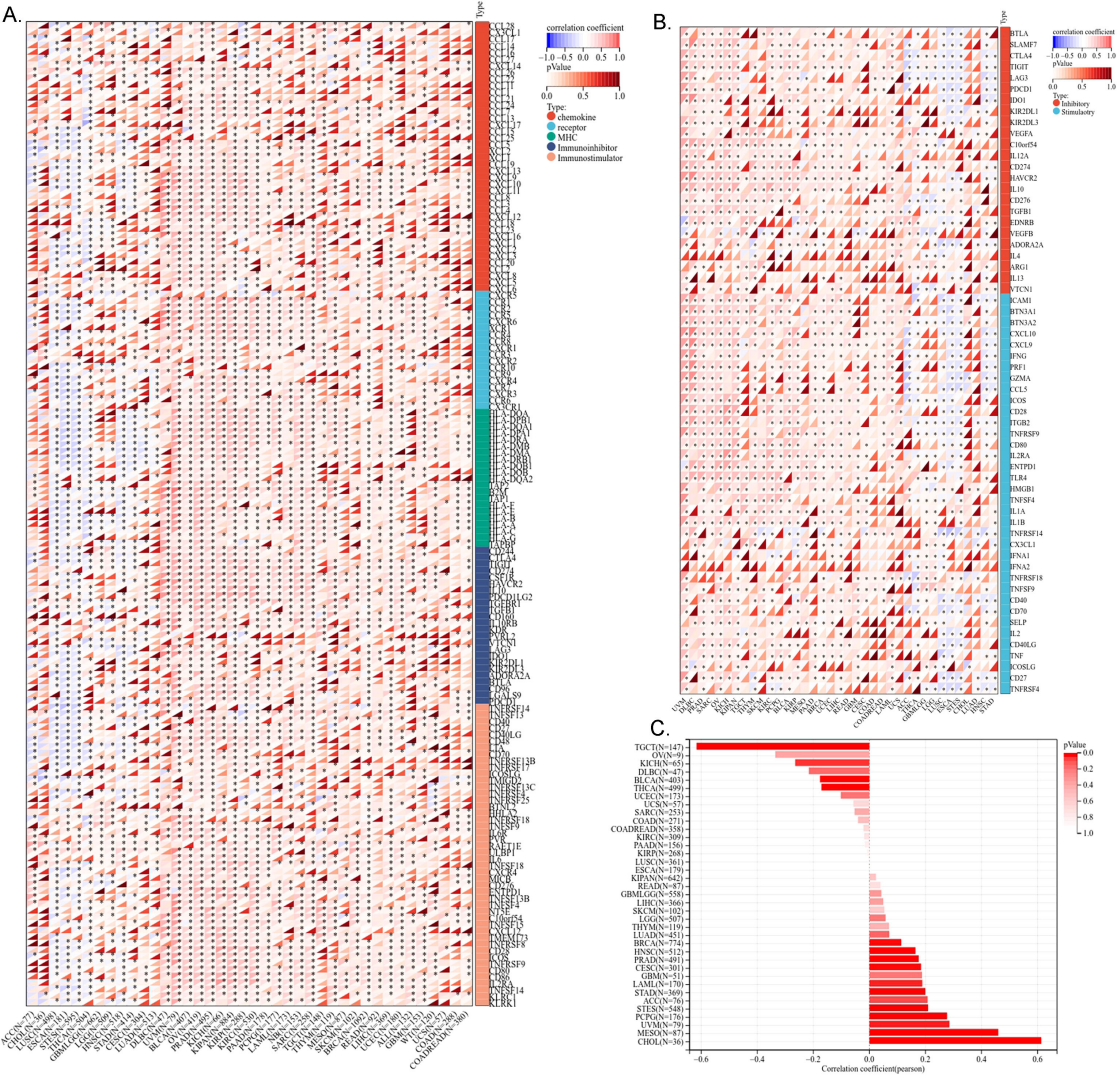
Correlation analysis of ME1 and immune regulatory gene, immune checkpoints and tumor stemness score. **(A)** The correlation of ME1 expression with most immune regulatory gene. **(B)** The correlation of ME1 and known immune checkpoints across all TCGA cancers. **(C)** The ME1 tumor stemness score. *P < 0.05.

We investigated the immune regulatory role of ME1 in tumors by analyzing its correlation with immune regulatory genes, immune checkpoints, tumor stemness scores, and immune infiltration. We utilized a heat map to visualize the correlation between the ME1 gene and five immune pathways: chemokines, receptors, MHC, immunosuppressants, and immune stimulators. The abscissa represents various cancers, while the ordinate denotes immune-related regulatory genes. The results indicated that the expression of ME1 was positively correlated with the majority of immune regulatory genes (**Figure 6A**). We subsequently analyzed the correlation between ME1 and various immune checkpoints, both inhibitory and stimulatory. The results from the heat map indicated a strong correlation between ME1 and the majority of immune checkpoints (**Figure 6B**). In our analysis of tumor stemness, we identified a significant association between the ME1 gene and tumor stemness scores across 14 tumor types. Notably, 11 of these tumor types (CHOL, mesothelioma [MESO], uveal melanoma [UVM], PCPG, soft tissue sarcoma [STES], STAD, LAML, cervical squamous cell carcinoma [CESC], PRAD, HNSC, and BRCA) exhibited a significant positive correlation with ME1, while a significant negative correlation was observed in 3 tumor types (THCA, BLCA, and TGCT) (**Figure 6C**).

#### Immune cell infiltration analysis of ME1

**Figure 7:**
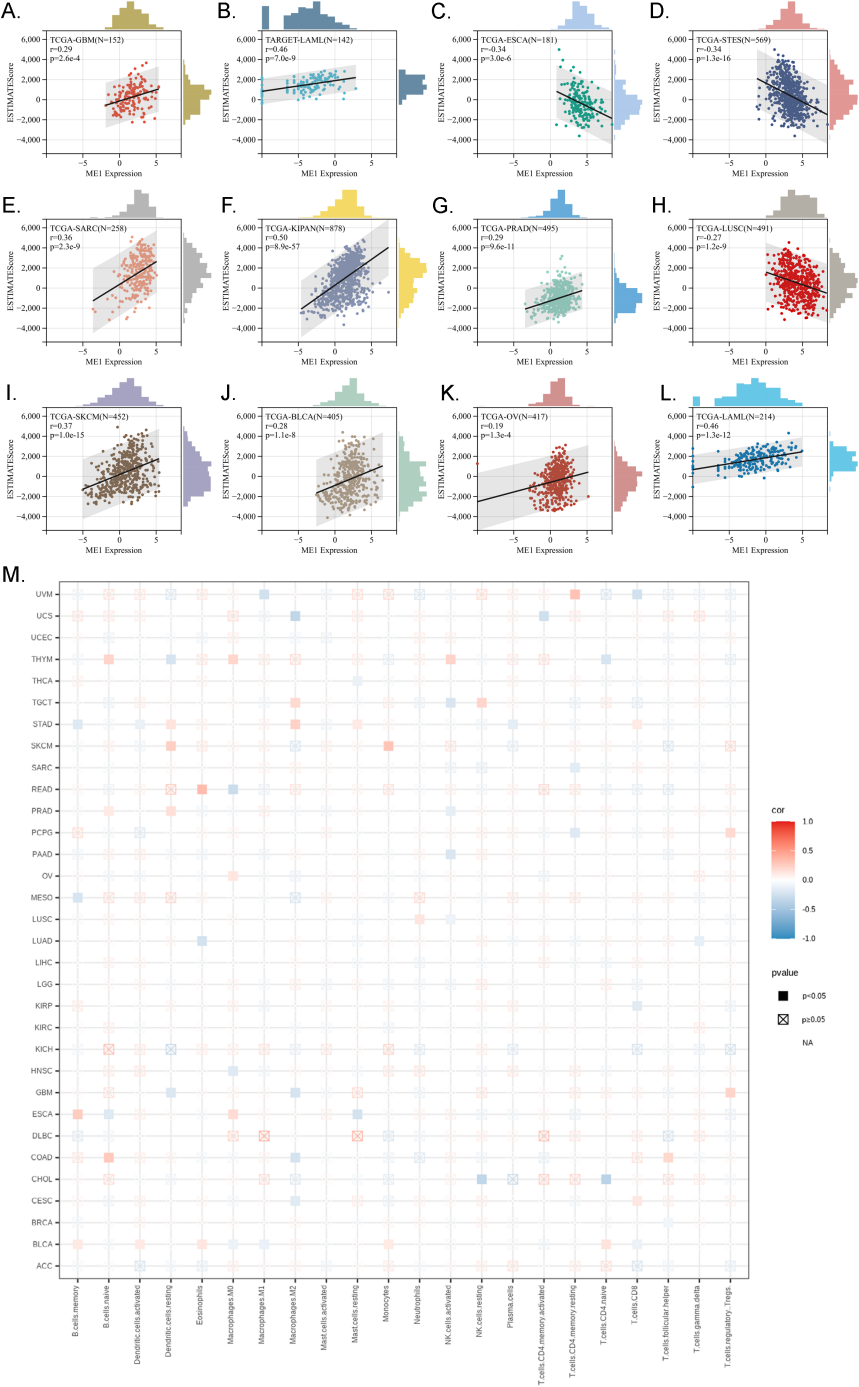
Immune infiltration analysis in pan-cancer. **(A-L)** Analysis of ME1 expression and immune infiltration score in tumor types. The results indicated that ME1 exhibited a significant positive correlation with immune infiltration in GBM, SARC, KIPAN, PRAD, SKCM, BLCA, LAML, and OV (*p* < 0.05). In contrast, in ESCA, STES, and LUSC, ME1 expression was negatively correlated with immune infiltration. **(M)** Examining the correlations between ME1expression and immune infiltration across all TCGA cancers using TIMER2 algorithms.

**Figure 8:**
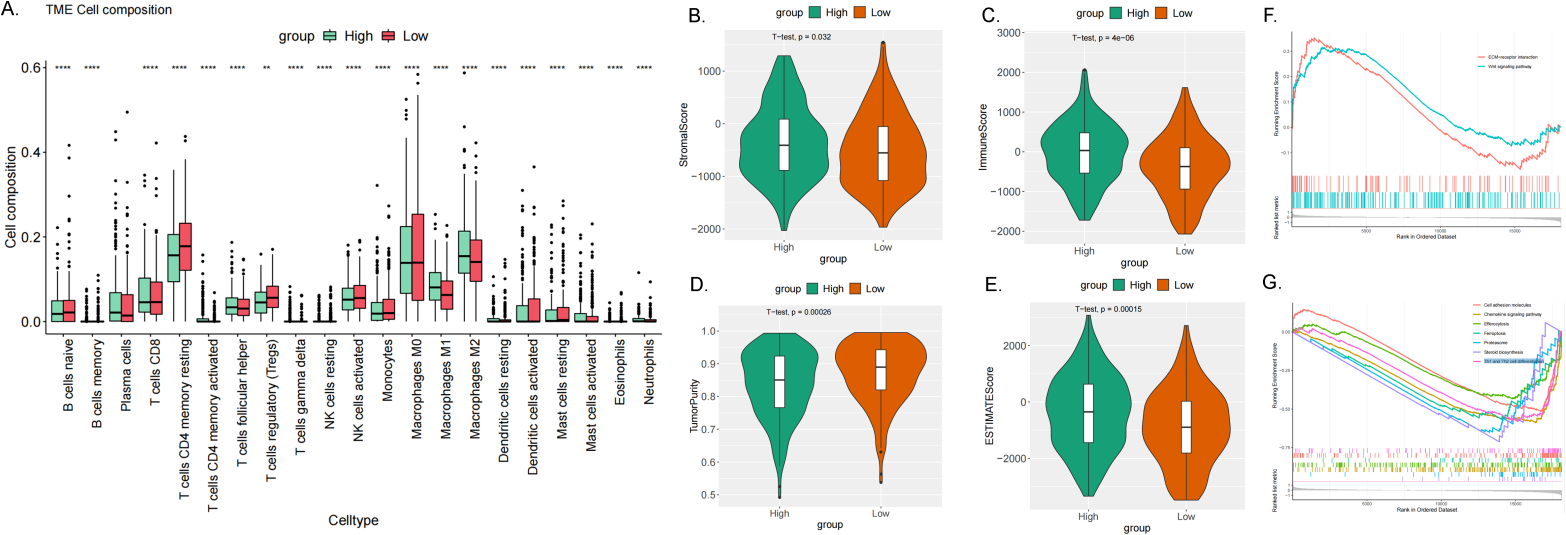
Immune infiltration analysis in OV. **(A)** The box plot illustrated the differences calculated by CIBERSORT in 22 types of immune infiltration cells between the high-risk and low-risk groups in OV. The statistical differences were shown as follow: **P <0.01; ****P <0.0001. **(B–E)** The violin plots showed the differences between high-risk and low-risk groups in stromal score **(B)**, immune score **(C)**, tumor purity **(D)** and estimated score **(E)** calculated using the ESTIMATE algorithm. **(F, G)** GSEA enrichment analysis revealed the biological functions and pathways in high-expression and low expression groups in ovarian cancer.

Immune infiltration analysis is central to the study of cancer immunotherapy and immune escape mechanisms, providing a theoretical foundation for precise treatment and disease prediction in clinical practice. The ESTIMATE score is a method used to analyze the infiltration of stromal and immune cells in tumor samples. It employs gene expression data to estimate the relative proportions of these cell types within the tumor microenvironment, serving as an important reference for tumor prognosis assessment, immunotherapy prediction, and molecular typing (26). We observed a significant correlation between the expression levels of the ME1 gene and tumor immune infiltration scores. ME1 exhibited a positive correlation with immune infiltration in several tumor types, including GBM, sarcoma (SARC), kidney renal papillary cell carcinoma (KIPAN), PRAD, skin cutaneous melanoma (SKCM), BLCA, LAML, and OV. These findings suggested that high expression levels of ME1 might facilitate the recruitment of immune cells into the tumor microenvironment. Conversely, a negative correlation between ME1 and immune infiltration was noted in ESCA, STES, and LUSC. This negative correlation might be attributed to the suppression of immune cell activity caused by ME1-driven metabolic reprogramming in these tumors (**Figures 7A-L**). Furthermore, we utilized TIMER2 to investigate the relationship between ME1 expression and the levels of various immune cell infiltrates across all TCGA tumors (**Figure 7M**) These results suggested that ME1 might influence the heterogeneity of the tumor immune microenvironment by regulating the recruitment and function of specific immune cell subsets, thereby impacting the efficacy of immunotherapy. However, further in-depth mechanistic experiments are required for validation. To further explore the potential signaling pathways linking ME1 to immune infiltration, we classified OV patients into high-expression and low-expression groups based on the median expression level of ME1. A box plot was used to illustrate the differences in immune cell infiltration within the tumor microenvironment between these groups (**Figure 8A**). The high-expression group demonstrated elevated levels of plasma cells, follicular helper T cells, and M1/M2 macrophages. Additionally, ESTIMATE analysis indicated a higher stromal score, immune score, and estimate score in the high-expression group, alongside increased tumor purity in the low-expression group (**Figures 8B-E**). The GSEA functional enrichment analysis identified significant enrichment of pathways related to ‘ECM−receptor interaction’ and ‘Wnt signaling pathway’ in the high-expression group (**Figure 8F**). Conversely, the low-expression group exhibited enrichment in pathways including ‘Cell adhesion molecules’, ‘Chemokine signaling pathway’, ‘Efferocytosis’, ‘Ferroptosis’, ‘Proteasome’, ‘Steroid biosynthesis’, and ‘Th1 and Th2 cell differentiation’ (**Figure 8G**).

#### The function analysis of ME1 in OV

**Figure 9:**
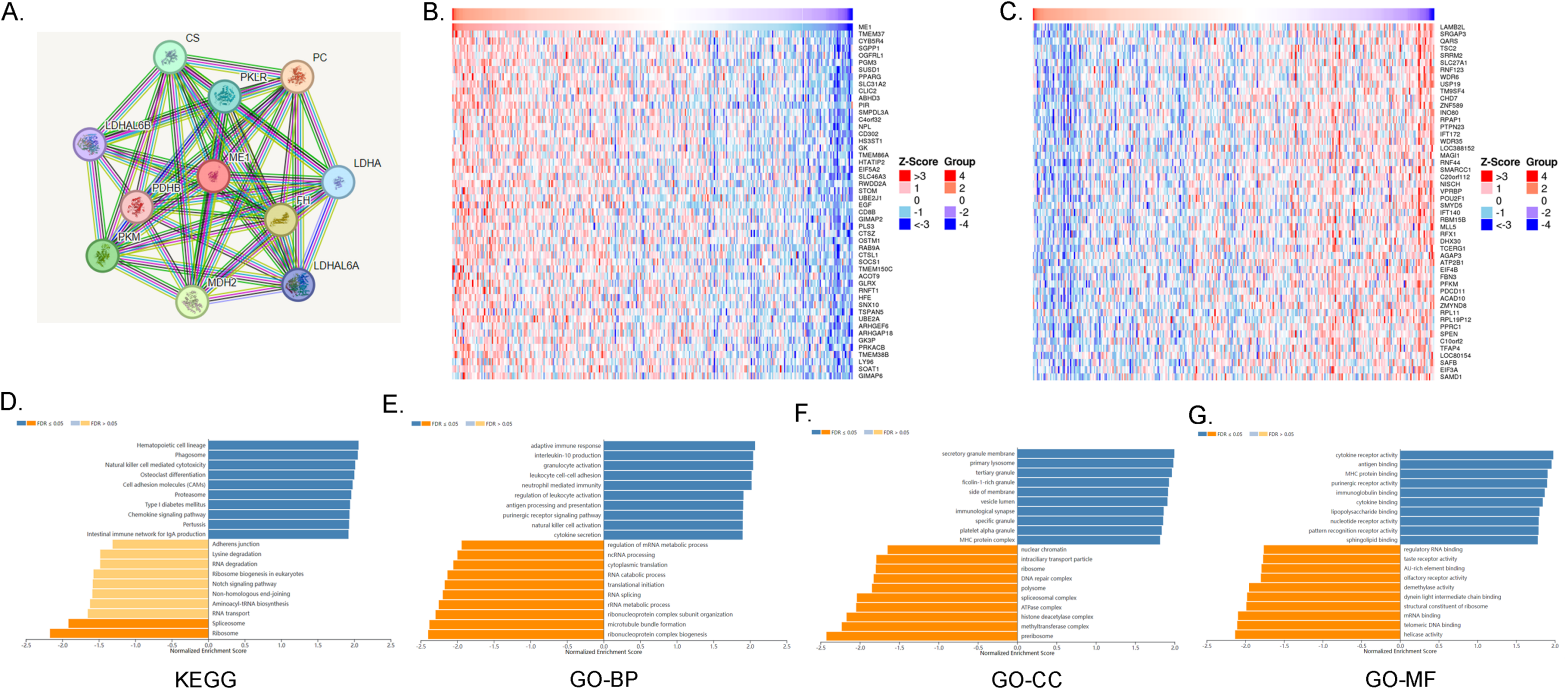
ME1 Functional enrichment analysis. **(A)**The protein-protein interaction network of ME1 from STRING. **(B)** The top 50 genes positively correlated to ME1 in the OV cohort. **(C)** The top 50 genes negatively correlated to ME1 in OV. **(D)** KEGG analysis of ME1 co-expression genes in the OV cohort. **(E-G)** GO analysis (BP, CC, and MF) of ME1 co-expression genes in the OV cohort.

The protein-protein interaction networks of ME1 were visualized using the STRING online database (**Figure 9A**). In ovarian cancer, the top 50 positively and negatively correlated genes associated with ME1 were further analyzed and presented in a heat map (**Figures 9B, C**). To elucidate the biological functions of ME1 in ovarian cancer, we conducted gene enrichment analysis using the LinkedOmics database. The KEGG analysis indicated significant enrichment in pathways related to hematopoietic cell lineage, phagosome activity, and natural killer cell-mediated cytotoxicity, among others (**Figure 9D**). The Gene Ontology categorizes gene functions into three distinct categories: cellular component (CC), molecular function (MF), and biological process (BP). GO analysis revealed that ME1 is primarily associated with adaptive immune response, interleukin-10 production, secretory granule membranes, primary lysosomes, cytokine receptor activity, and antigen binding, among other functions (**Figures 9E-G**). This study found that ME1 is enriched in immune-related pathways, suggesting that as a metabolism-related molecule, it may play a role in the metabolic-immune interactions involved in the development and progression of tumors. The underlying mechanisms warrant further in-depth investigation.

#### Drug sensitivity and the immunotherapy analysis of ME1 in OV

**Figure 10:**
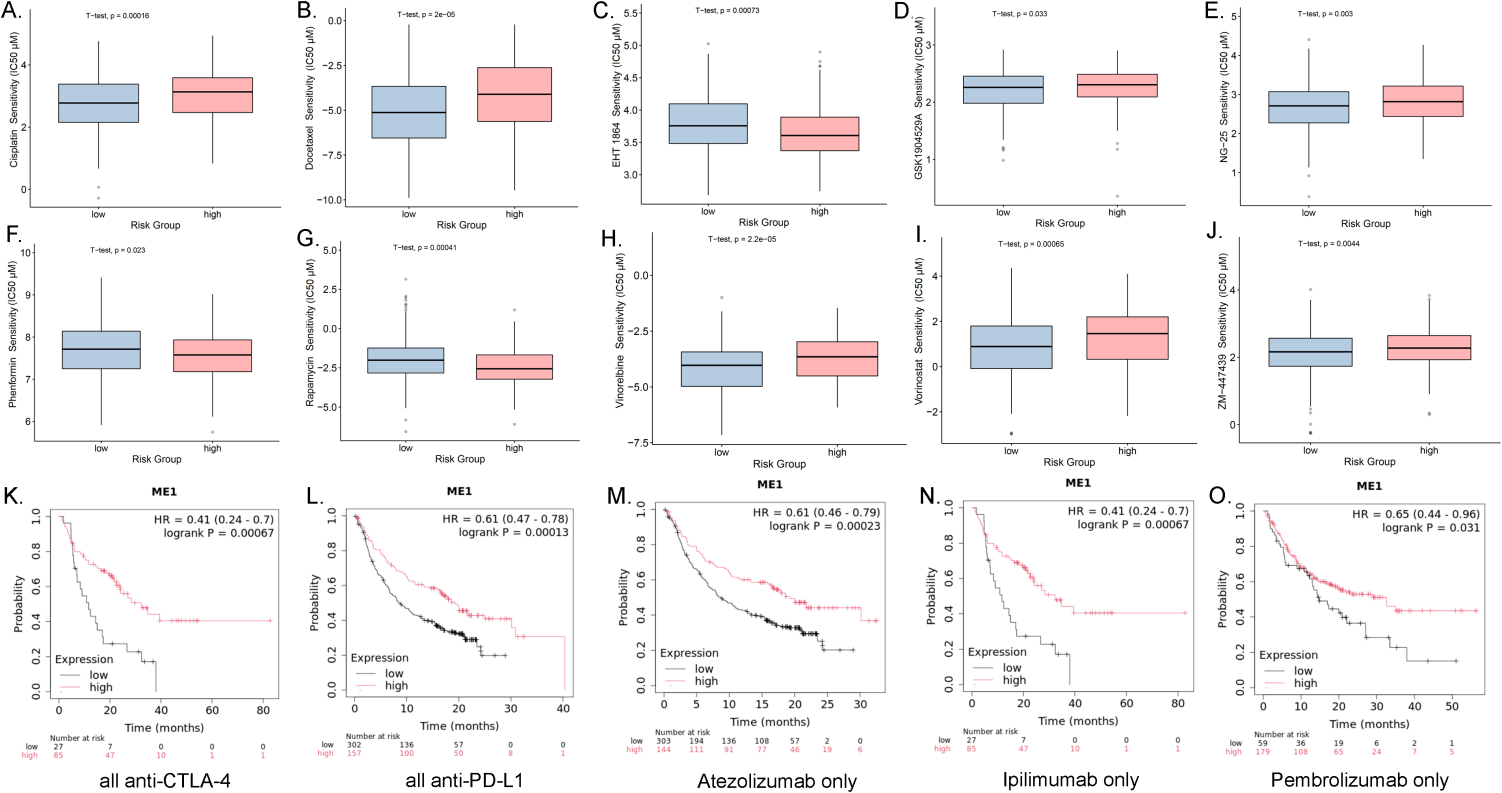
Drug sensitivity analysis and the associations between ME1 expression and immune checkpoint blockade treatment in cancer. **(A-J)** The box plots of the estimated IC50 displayed the differences of drug sensitivity between the high-risk and low-risk groups in the OV cohort. **(K)** Overall survival using all anti-CTLA-4 between the high-risk and low-risk groups in the OV cohort. **(L)** Overall survival using all anti-PD-L1 between the high-risk and low-risk groups in the OV cohort. **(M)** Overall survival using all Atezolizumab only between the high-risk and low-risk groups in the OV cohort. **(N)** Overall survival using all Ipilimumab only between the high-risk and low-risk groups in the OV cohort. **(O)** Overall survival using all Pembrolizumab only between the high-risk and low-risk groups in the OV cohort.

We categorized the ME1 expression values of ovarian cancer patients into high and low expression groups based on the median. Utilizing the GDSC database, we conducted a sensitivity analysis on chemotherapy drugs and compared the half-maximum inhibitory concentration (IC50) values between the high-risk and low-risk groups. The study revealed that EHT1864, phenformin, and rapamycin exhibited lower IC50 values (μM) in the high-risk group, whereas cisplatin, docetaxel, GSK1904529A, NG-25, vinorelbine, vorinostat, and ZM-447439 demonstrated higher IC50 values in the high-risk group (**Figures 10A-J**). In addition, to investigate the potential of ME1 as a target for tumor immunotherapy, we analyzed the overall survival rates of patients exhibiting high versus low expression of ME1 following treatment with anti-PD1, anti-PDL1, or anti-CTLA4 therapies. Patients who received immunotherapy with Atezolizumab alone, any anti-CTLA-4 agent, any anti-PD-L1 agent, Pembrolizumab alone, or Ipilimumab alone exhibited significantly improved overall survival when they had higher ME1 expression compared to those with lower ME1 expression (**Figures 10K-O**). This finding suggested that elevated levels of ME1 expression might correlate with a more favorable response to specific immunotherapies. Further in-depth research into the mechanisms underlying drug sensitivity is necessary for validation.

### Functional validation of ME1 in OV

#### ME1 knockdown inhibited the malignant behaviors in OV cells

**Figure 11:**
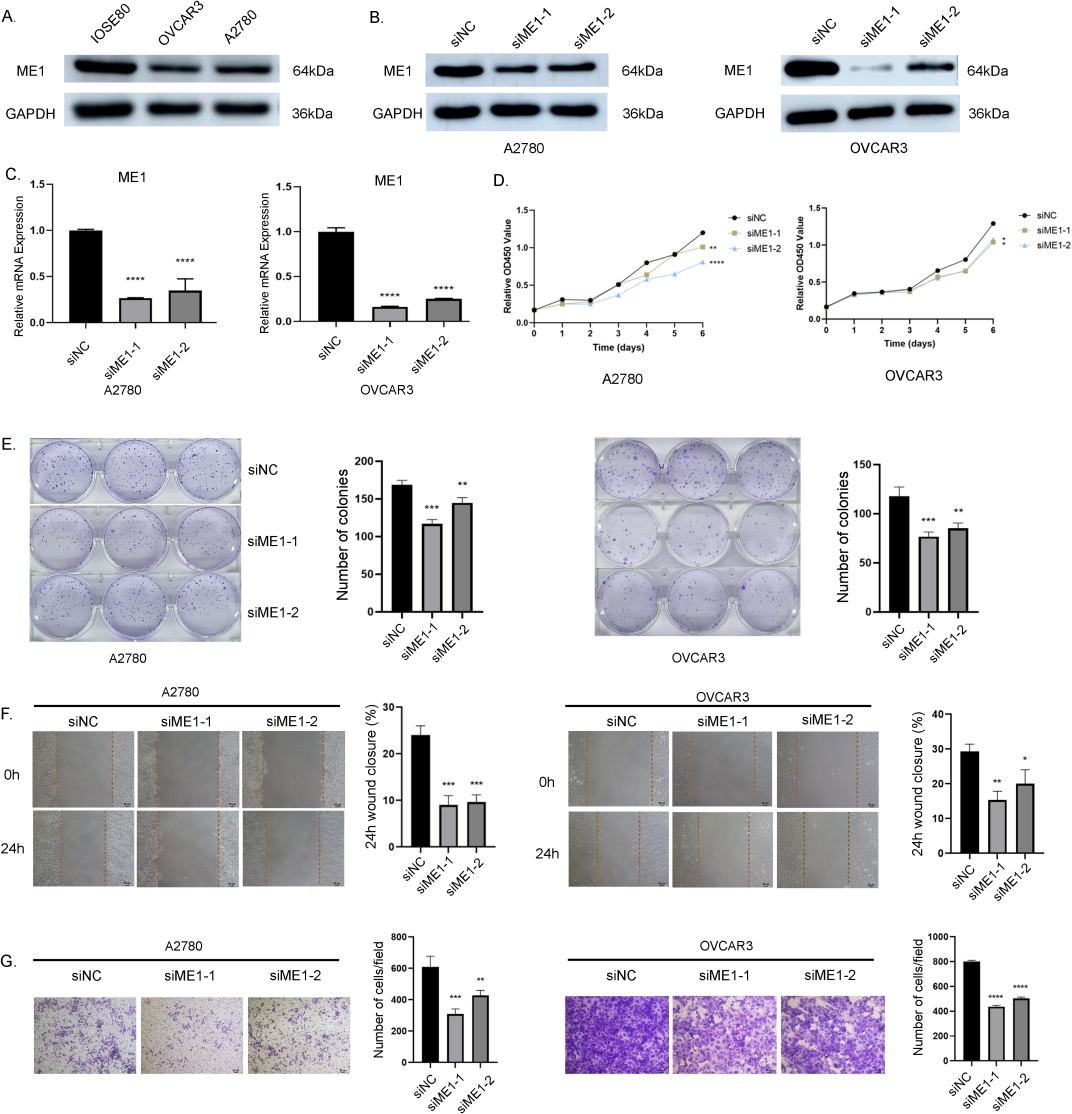
ME1 knockdown suppressed the proliferation and migration in OV cell lines. **(A)** ME1 basal expression in normal ovarian cells (IOSE80) and in human ovarian cancer cell lines (A2780, and OVCAR3). **(B, C)** WB and qRT-PCR results confirmed the effective knockdown of the ME1 gene mediated by siRNA in the A2780 and OVCAR3 cell lines, respectively. **(D)** CCK-8 assay results indicated that the knockdown of the ME1 gene inhibited the proliferative capacity of both A2780 and OVCAR3 cell lines. **(E)** Clone formation assay further corroborated that ME1 knockdown reduced the proliferative ability of A2780 and OVCAR3 cells. **(F)** Cell scratch assays revealed a significant decrease in the migration of A2780 and OVCAR3 cells following ME1 knockdown. **(G)** Transwell assays demonstrated that silencing ME1 markedly impaired the migratory capability of A2780 and OVCAR3 cells.*p < 0.05, **p < 0.01, ***p < 0.001, ****p < 0.0001.

We assessed the basal expression levels of ME1 in normal ovarian cells (IOSE80) and in human ovarian cancer cell lines (A2780, and OVCAR3) using Western blotting (WB) to analyze protein expression levels (**Figure 11A**). The findings indicated that ME1 was expressed at lower levels in human ovarian cancer cell lines compared to normal ovarian cells. We identified two effective ME1 siRNA primer sequences and assessed the knockdown efficiency of ME1 through qRT-PCR and WB in human ovarian cancer cell lines A2780 and OVCAR3 (**Figures 11B, C**). Following the successful knockdown of ME1, we conducted CCK8 and clone formation assays to assess the proliferation capacity of the cells (**Figures 11D, E**). The results indicated that the knockdown of ME1 significantly reduced the proliferation ability of the A2780 and OVCAR3 cell lines. Furthermore, both cell scratch and transwell assays demonstrated that ME1 silencing markedly impaired the migration capability of A2780 and OVCAR3 cells (**Figures 11F, G**).

#### ME1 overexpression promoted aggressive behaviors in OV cells

**Figure 12:**
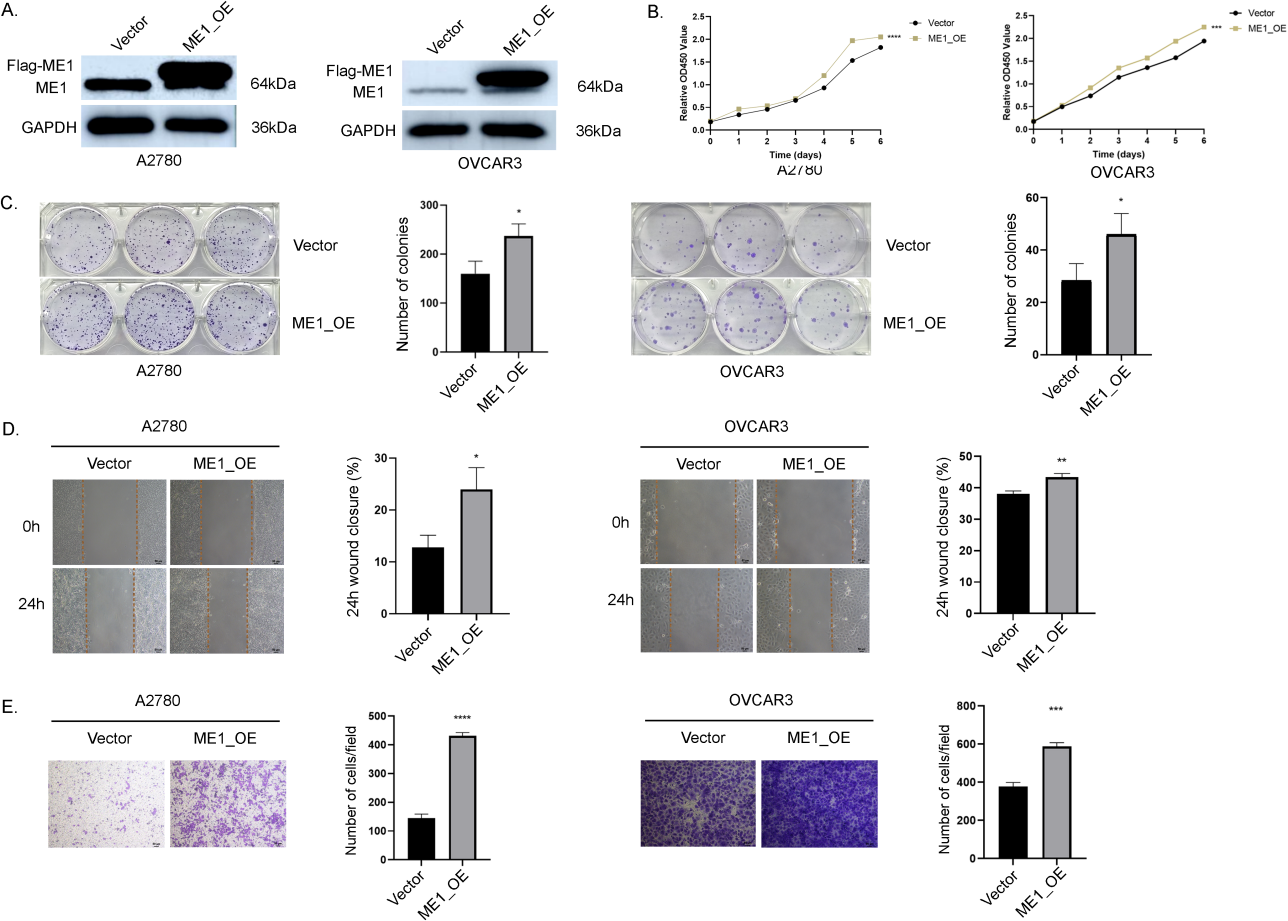
ME1 overexpression promoted the proliferation and migration in OV cell lines. **(A)** WB analysis of ME1 protein expression was conducted in vector-control cells (vector) and ME1-overexpressing cells (ME1_OE). **(B, C)** The overexpression of ME1 significantly enhanced proliferative activity, as demonstrated by the CCK-8 assay and colony formation assay. **(D, E)** Both the cell scratch assay and transwell migration assay indicated that ME1 overexpression resulted in an increased number of migrating cells. *P < 0.05; **P < 0.01; ***P < 0.001; ****P < 0.0001.

To further investigate the critical role of ME1 in OV, we transduced the human ovarian cancer cell lines A2780 and OVCAR3 with a ME1-overexpressing lentiviral and a negative control vector. WB analysis confirmed a significant increase in ME1 expression in both A2780 and OVCAR3 cells (**Figure 12A**). Results from both the clone formation assays indicated that the proliferation capacity of A2780 and OVCAR3 cells was markedly enhanced following ME1 overexpression (**Figures 12B, C**). Additionally, cell migration assays, including the cell scratch and transwell assays, demonstrated that the migratory ability of A2780 and OVCAR3 cells with ME1 overexpression was significantly increased compared to the negative control (**Figures 12D, E**).

## Discussion

Cancer is a complex disease characterized by abnormal cell growth and division. Its etiology involves a multifaceted interplay of genetic, environmental, lifestyle, and other factors (28). With the advent of personalized and precise diagnostic and therapeutic approaches, it is essential to conduct a comprehensive analysis of each patient’s unique circumstances, including, but not limited to, the pathological type and stage of cancer, as well as the patient’s overall health status (22). Pan-cancer analysis serves as a valuable component of tumor bioinformatics and plays a crucial role in the personalized diagnosis and treatment of cancer patients (3). In this study, we conducted a comprehensive bioinformatics analysis of ME1 across various cancer types, focusing on its expression levels, mutations, methylation levels, immune infiltration, and potential functional roles. We analyzed and verified the expression of ME1 in both tumor and normal tissues at the mRNA and protein levels. These results indicated that, at the mRNA level, ME1 expression was reduced in 16 types of cancer and increased in 4 types, with all findings reaching statistical significance. Furthermore, analyses of protein expression and immunohistochemistry confirmed that ME1 was differentially expressed across various tumors, suggesting that the dysregulation of ME1 may contribute to both the occurrence and progression of tumors. Our pan-cancer analysis revealed that ME1 expression varies across different tumor types, and this heterogeneity reflects its context-dependent functions in tumor biology. In tumors reliant on lipid synthesis, high ME1 expression supports NADPH and fatty acid production. Conversely, in glycolysis-dominant or hypoxic microenvironments, low ME1 expression may represent an adaptive strategy, illustrating its “dualistic” functionality. This variability may arise from metabolic heterogeneity, epigenetic regulation, mutational burden, and interactions with the immune microenvironment. Future research should focus on elucidating the tissue-specific regulatory networks of ME1, conducting mechanistic studies, integrating multi-omics data, and performing preclinical trials to optimize precision therapeutic strategies.

Gene mutations and methylation modifications are recognized as key factors that lead to the dysregulation of gene expression in cancer, consequently influencing both the occurrence and progression of the disease (29). This study identifies missense mutations as the predominant form of gene mutation in ME1. Additionally, it highlights the close relationship between MEI and genes associated with DNA and RNA methylation. Currently, the molecular mechanism of ME1 has primarily been investigated in specific types of cancer, leaving its role in pan-cancer largely unclear. In this study, we conducted a gene enrichment analysis and single-cell analysis of ME1. The results preliminarily revealed correlations between ME1 and macrophages, natural killer cell-mediated cytotoxicity, the adaptive immune response, interleukin-10 production, and cytokine receptor activity. We infer that ME1 may regulate the metabolic state of T cells through NADPH-dependent redox homeostasis, thereby affecting their activation and anti-tumor functions. It modulates the functions of secretory granule membranes in cytotoxic T cells and NK cells, influencing the efficiency of perforin and granzyme release, which compromises the tumor cell lysis capability. IL-10, a pleiotropic cytokine secreted by regulatory T cells (Tregs), M2 macrophages, and tumor cells, plays a dual role in the tumor microenvironment. On one hand, it mitigates immune damage by suppressing pro-inflammatory factors such as TNF-α and IL-12; on the other hand, it fosters an immunosuppressive microenvironment by inhibiting effector T cell activity, promoting Treg expansion, and polarizing M2 macrophages, thereby mediating tumor immune evasion (30). The results indicate that ME1, as a metabolic hub, may drive immune escape in ovarian cancer through multidimensional interactions involving redox balance, secretory pathways, and cytokine signaling. Specific molecular mechanisms require further experimental validation. Macrophages are essential components of the innate immune system. The activation of macrophages initiates multiple signaling pathways and is closely associated with metabolic changes, which in turn drive the differentiation of various immune subpopulations (31). Anna Santarsiero et al. found that ME1 was overexpressed in lipopolysaccharide-induced macrophages and was regulated by the NF-κB pathway. Silencing the ME1 gene resulted in a reduction in the production of nitric oxide, reactive oxygen species, and prostaglandin E2, which are key inflammatory mediators (14). We hypothesize that ME1 may participate in the tumor immune microenvironment through metabolic-immune crosstalk and the regulation of immune checkpoints. Future research should employ conditional knockout models or single-cell metabolomics to elucidate the specific mechanisms by which ME1 contributes to tumor immune evasion.

ME1 plays a significant role in regulating the supply of NADPH, oxidative stress, lipid metabolism, and immunosuppression within the tumor microenvironment through metabolic reprogramming. ME1 facilitates ATP production for vascular endothelial cells via glycolysis and the TCA cycle, thereby promoting their proliferation and migration. As a cofactor for endothelial nitric oxide synthase (eNOS), NADPH allows ME1 to influence vasodilation and neovascularization by modulating nitric oxide (NO) levels. Furthermore, ME1 may indirectly participate in angiogenesis and stromal cell interactions through the regulation of growth factors (9). Cancer develops within a complex tissue environment, where bidirectional communication between cells and their microenvironment is essential for maintaining normal tissue homeostasis and promoting tumor growth. Notably, the interactions between tumor cells and the surrounding stroma significantly influence disease onset, progression, and patient prognosis (32). In this study, we identified intriguing associations between ME1 expression and TMB, MSI, and tumor stemness. Both TMB and MSI enhance immunogenicity, thereby improving responses to immune checkpoint inhibitors (33). Our analysis revealed that ME1 expression exhibits both positive and negative correlations with TMB, MSI, and tumor stemness status, with variations observed across different cancer types. The cancer-specific nature of these associations indicates complex, context-dependent interactions between ME1 and the tumor immune microenvironment. By influencing TMB, MSI, and tumor differentiation, ME1 may play a regulatory role in anti-tumor immune responses. Uncontrolled cell proliferation is a hallmark of malignant tumors. Immunotherapy is increasingly favored over other cancer therapies due to its capacity to precisely target malignant cells while also enhancing the intricate responses of the immune system. This approach is closely related to a comprehensive understanding of tumor biology, particularly the complex interactions among tumor cells, the immune system, and the TME (34). In this study, we evaluated the role of ME1 in immune response from various dimensions. By analyzing the relationship between ME1 expression and immune regulation genes, immune examination points, and immune infiltration, we revealed the significant role of ME1 in the tumor microenvironment. ME1 exhibited a significant correlation with immunochemical checkpoints, immunomodulatory regulators, immunomodulatory regulatory genes, cell functional status, and immune infiltration. This suggests that ME1 may play a pivotal role in tumor development by modulating the immune response and promoting tumor invasion.

Previous studies have demonstrated that ME1 functions as a cancer-promoting gene. The suppression of ME1 gene expression is associated with a reduction in the transformation and migration of epithelial cells, while simultaneously promoting oxidative stress and apoptosis in tumor cells (13). The cell experiments conducted in this study provide direct evidence elucidating the role of ME1 in ovarian cancer cell lines. We initially employed WB and qRT-PCR to assess the baseline expression of ME1 in normal ovarian cells as well as in ovarian cancer cell lines A2780 and OVCAR3, which aligns with the findings from our biological information analysis. Additionally, we observed that reducing ME1 expression led to a decrease in the proliferation and migration capabilities of ovarian cancer cells. Conversely, increasing ME1 expression enhanced these capacities, suggesting that ME1 may facilitate the proliferation and metastasis of tumor cells, thereby promoting cancer progression. Although this study systematically revealed the relationship between ME1 and various types of cancer, it still has certain limitations. Firstly, We reviewed the existing literature and found that ME1, a key metabolic gene, is associated with various cancers. However, most of these studies are either fundamental or preclinical, and to date, there have been no clinical trial reports directly targeting ME1. Mechanistically, ME1 links glycolysis, the TCA cycle, and the generation of NADPH, which are crucial for maintaining redox balance, lipid synthesis, and immune cell function within the tumor microenvironment. These pathways align with established metabolic targets in oncology, such as IDH1/2 and FASN, some of which have progressed to clinical trials. Currently, therapies targeting related metabolic pathways are under clinical investigation. In the future, we will focus more on conducting ME1-related clinical trials to establish a foundation for the translation of basic research into clinical practice. Secondly, due to the current limitations in the accessibility of clinical samples, this study did not encompass all subtypes and stages of ovarian cancer. We are currently in the process of collecting tissue specimens from ovarian cancer patients in our hospital. Furthermore, for certain rare pathological types, we plan to collaborate with multiple centers in the future to expand the sample size and include these rare subtypes. By integrating single-cell sequencing technology, we aim to analyze the cell type-specific expression patterns of ME1 within the tumor microenvironment. Existing literature has documented metabolic differences among various subtypes of ovarian cancer. The current experiments are primarily based on cell models. In the future, our research group will validate the stage-specific regulatory mechanisms of ME1 using mouse models and multicenter clinical immunotherapy cohorts, and will explore its potential as a marker for subtype stratification. Thirdly, The *in vitro* experiments conducted in this study focused on the impact of ME1 on the autonomous behavior of tumor cells; however, the efficacy of immunotherapy involves complex tumor-immune interactions. The expression of ME1 in immune cells and its regulation of immune metabolism may be crucial for elucidating its clinical relevance. Subsequent studies will integrate immunodeficient mouse models, immune cell co-culture experiments, and single-cell sequencing technologies to clarify the specific functions of ME1 across different cell types. The *in vitro* experiments conducted in this study primarily focus on the impact of ME1 on the functional phenotypes of tumor cells. The internal dynamic regulatory mechanisms and the efficacy of immunotherapy involve complex interactions among tumor metabolism and the immune system. Thus, the dynamic regulation of ME1 and its modulation of immunometabolism may be crucial for elucidating its clinical significance. Future research will involve more comprehensive and in-depth *in vitro* mechanistic experiments, including immune cell co-culture experiments, single-cell sequencing technology, pathway validation, and metabolite detection. Additionally, *in vivo* animal models, such as immunodeficient mouse models, and clinical trials will be employed to further explore its dynamic changes and regulation, which will also guide the direction of our future research.

## Conclusions

In summary, ME1 catalyzes the transformation of apple acid into pyruvic acid, which serves as a link between glucose and citric acid. In this study, we conducted a preliminary analysis of ME1 expression across various cancers, focusing on its expression patterns and fundamental biological characteristics in tumors, particularly in relation to the immune system. ME1 appears to play a crucial role in facilitating immune cell infiltration into the tumor microenvironment. Furthermore, we confirmed that high expression levels of ME1 promote the proliferation and migration of ovarian cancer cells. The immunoregulatory function of ME1, along with its association with TMB and MSI, suggests its potential as a predictive target for immunotherapy response. Further validation through more in-depth *in vivo* studies, animal models, and clinical trials, supported by multi-omics approaches, is needed to comprehensively elucidate the role of ME1 in ovarian cancer metabolism and immunity, thereby facilitating the translation from basic research to clinical application.

## Data Availability

The raw data supporting the conclusions of this article will be made available by the authors, without undue reservation.
