# Supplementary material for: The potential of ME1 in guiding immunotherapeutic strategies for ovarian cancer: insights from pan-cancer research
Source: Front Immunol. 2025 May 29;16:1571842. doi: 10.3389/fimmu.2025.1571842 (PMC12159071; doi:10.3389/fimmu.2025.1571842)
Supplement: Supplementary file 1 [file Table1.docx]

Supplementary table 1 the ME1 siRNA SYBR primer comparison table

| RNA name | Primer Sequence |
| --- | --- |
| siNC | 5′-UUCUCCGAACGUGUCACGUTT-3′  5′-ACGUGACACGUUCGGAGAATT-3′ |
| siME1-1 | 5′-GGUGCAUUCUCAGAACAAATT-3′  5′-UUUGUUCUGAGAAUGCACCTT-3′ |
| siME1-2 | 5ʹ-CCAGGUUCUUAGAGUAGUATT-3ʹ  5ʹ-UACUACUCUAAGAACCUGGTT-3ʹ |
