# Supplementary material for: The potential of ME1 in guiding immunotherapeutic strategies for ovarian cancer: insights from pan-cancer research
Source: Front Immunol. 2025 May 29;16:1571842. doi: 10.3389/fimmu.2025.1571842 (PMC12159071; doi:10.3389/fimmu.2025.1571842)

**ME1 basic expression**

ME1（Group from left to right: IOSE80-A2780-OVCAR3-SKOV3）


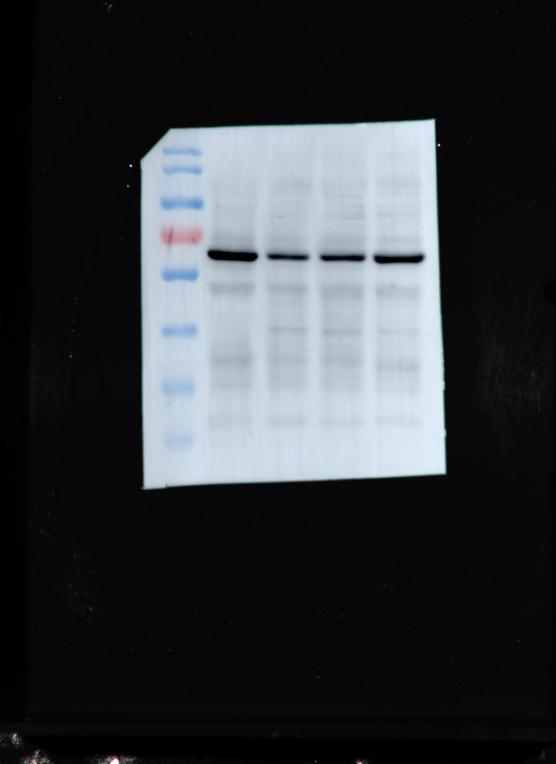


GAPDH(Group from left to right: IOSE80-A2780-OVCAR3-SKOV3)


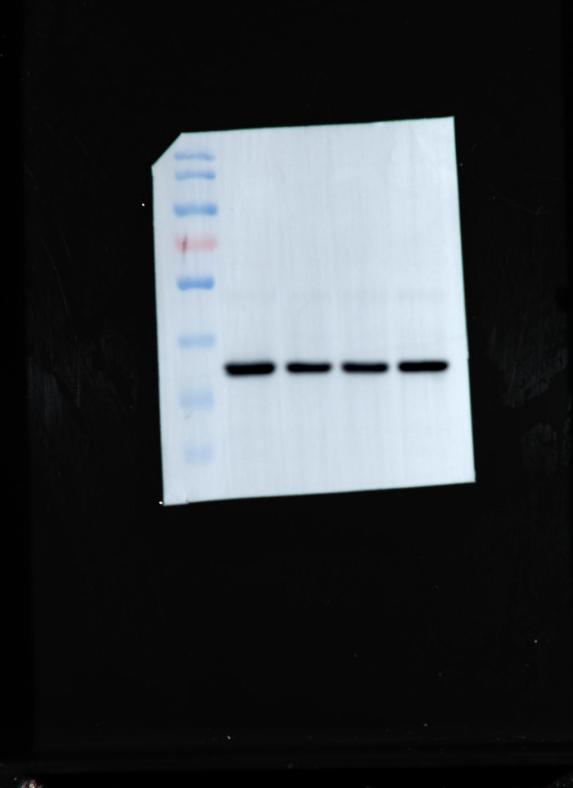


**ME1 knockdown efficiency verification**

ME1-A2780-siME1


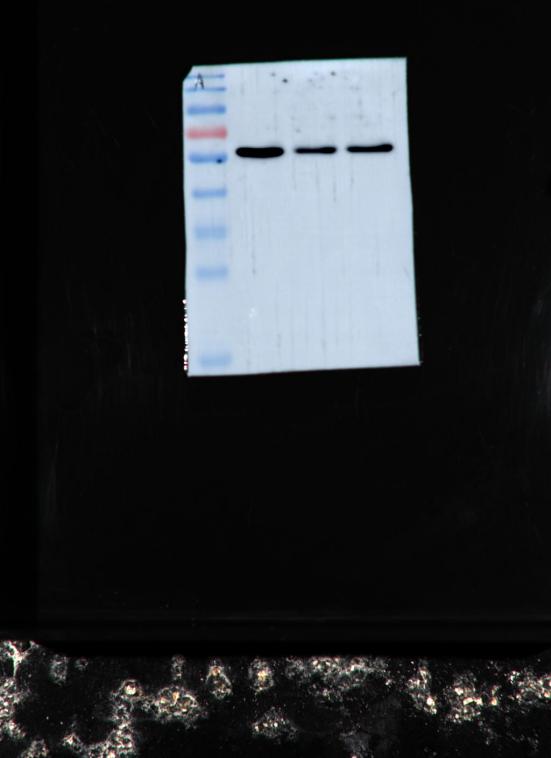


GAPDH-A2780-siME1


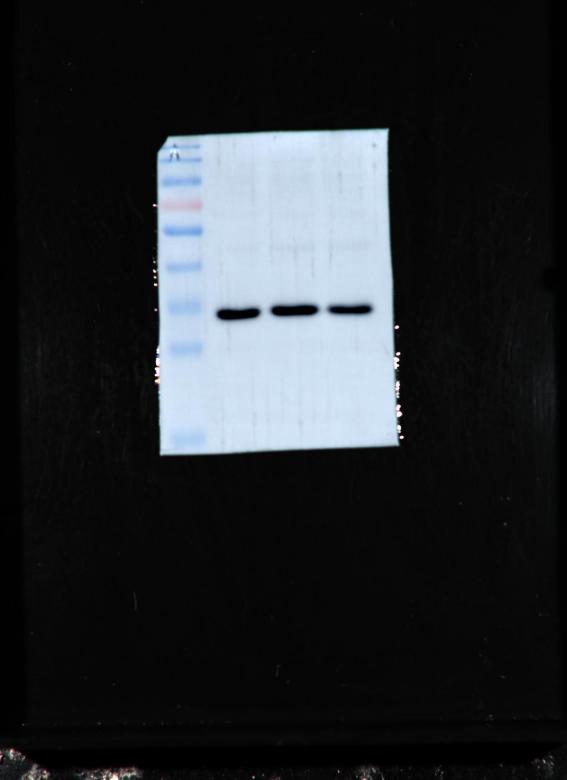


ME1-OVCAR3-siME1


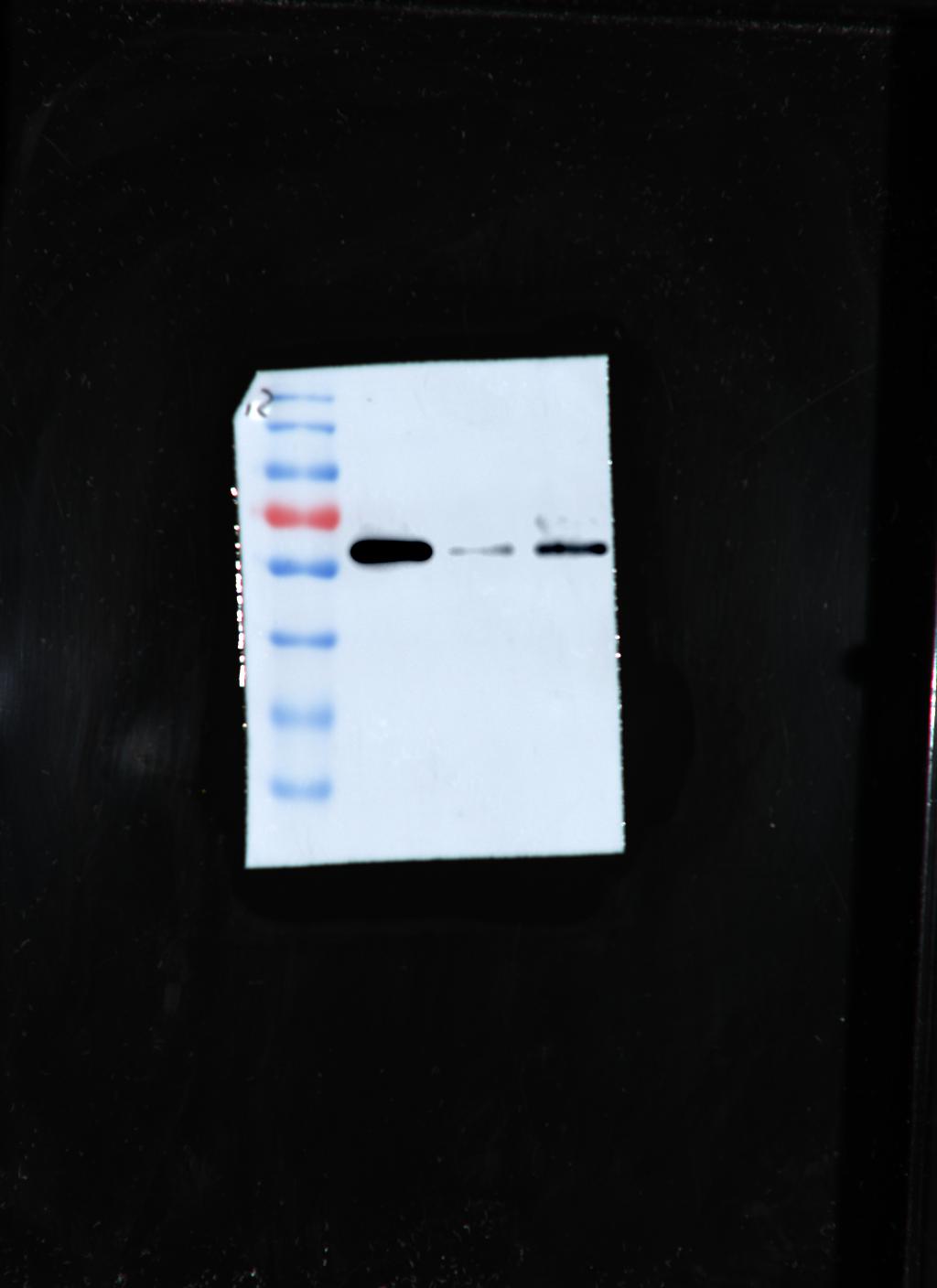


GAPDH-OVCAR3-siME1


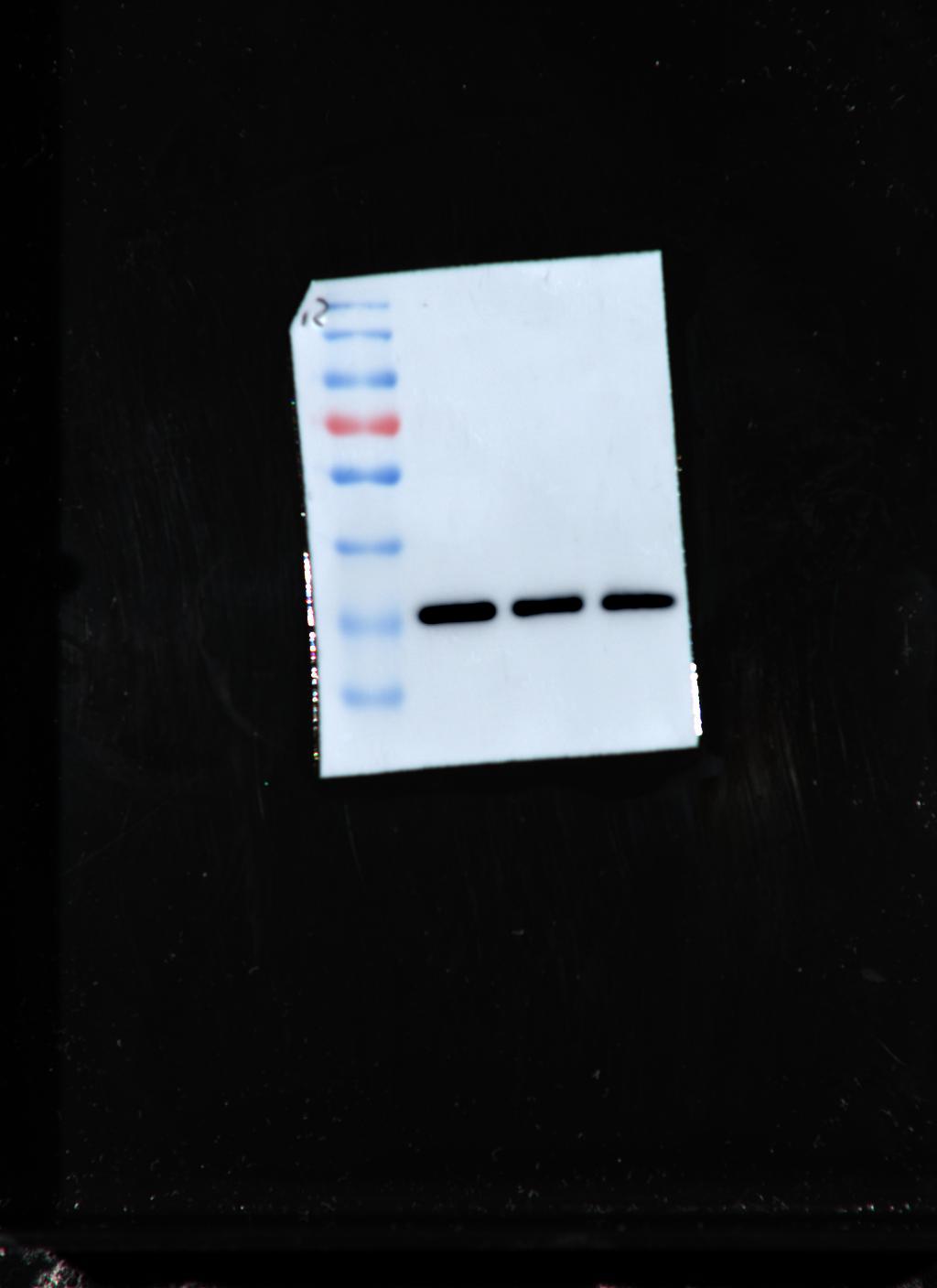


**ME1 overexpression efficiency verification**

ME1-A2780-ME1_OE


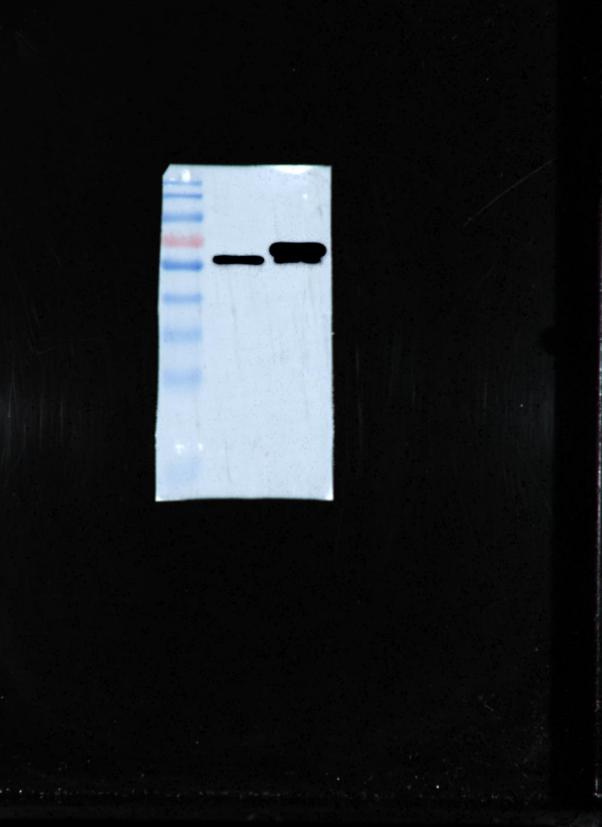


GAPDH-A2780-ME1_OE


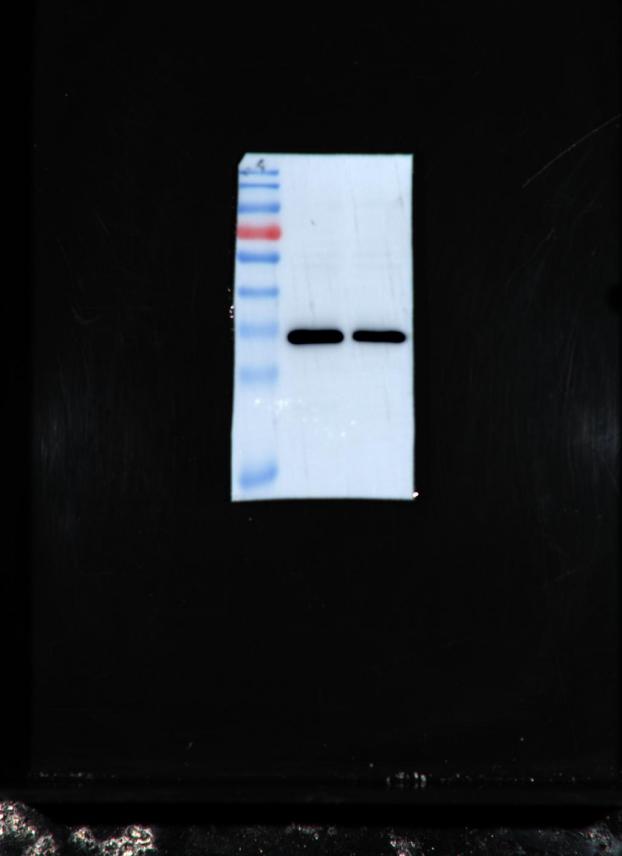


ME1-OVCAR3-ME1_OE


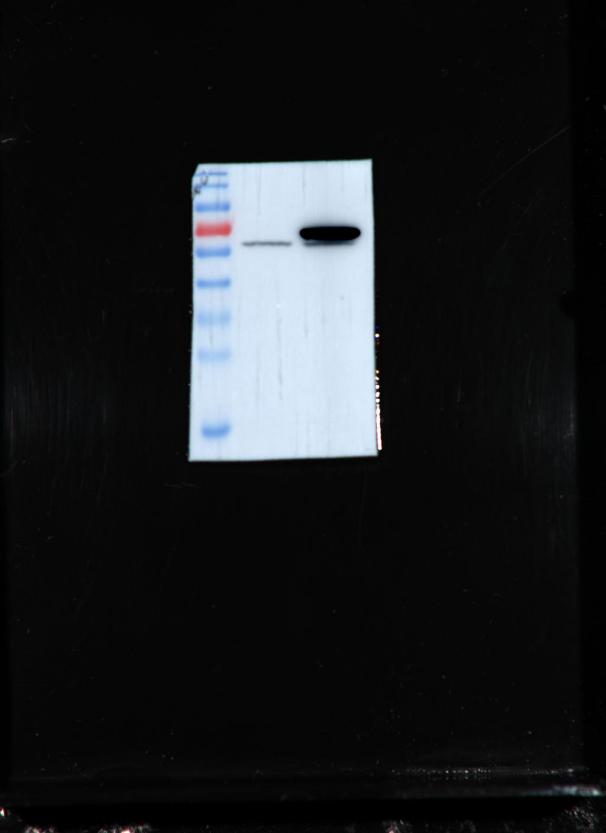


GAPDH-OVCAR3-ME1_OE


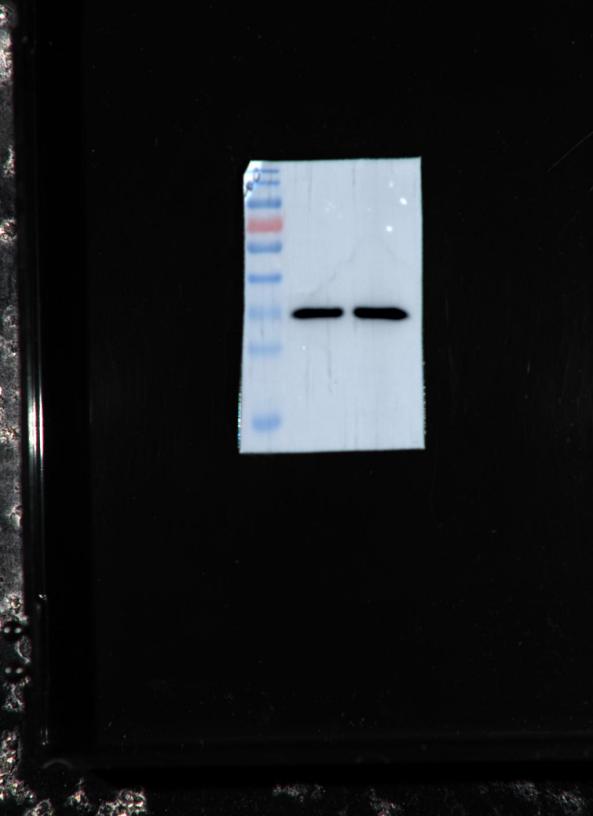

Supplement: Supplementary file 2 [file DataSheet1.docx]
